# Structural prerequisites for CRM1-dependent nuclear export signaling peptides: accessibility, adapting conformation, and the stability at the binding site

**DOI:** 10.1038/s41598-019-43004-0

**Published:** 2019-04-29

**Authors:** Yoonji Lee, Jimin Pei, Jordan M. Baumhardt, Yuh Min Chook, Nick V. Grishin

**Affiliations:** 1 Department of Biophysics, University of Texas Southwestern Medical Center, Dallas, TX 75390 USA; 2 Howard Hughes Medical Institute, University of Texas Southwestern Medical Center, Dallas, TX 75390 USA; 3 Department of Pharmacology, University of Texas Southwestern Medical Center, Dallas, TX 75390 USA

**Keywords:** Computational biophysics, Protein structure predictions, Computational models, Molecular modelling

## Abstract

Nuclear export signal (NES) motifs function as essential regulators of the subcellular location of proteins by interacting with the major nuclear exporter protein, CRM1. Prediction of NES is of great interest in many aspects of research including cancer, but currently available methods, which are mostly based on the sequence-based approaches, have been suffered from high false positive rates since the NES consensus patterns are quite commonly observed in protein sequences. Therefore, finding a feature that can distinguish real NES motifs from false positives is desired to improve the prediction power, but it is quite challenging when only using the sequence. Here, we provide a comprehensive table for the validated cargo proteins, containing the location of the NES consensus patterns with the disordered propensity plots, known protein domain information, and the predicted secondary structures. It could be useful for determining the most plausible NES region in the context of the whole protein sequence and suggests possibilities for some non-binders of the annotated regions. In addition, using the currently available crystal structures of CRM1 bound to various classes of NES peptides, we adopted, for the first time, the structure-based prediction of the NES motifs bound to the CRM1’s binding groove. Combining sequence-based and structure-based predictions, we suggest a novel and more straight-forward approach to identify CRM1-binding NES sequences by analysis of their structural prerequisites and energetic evaluation of the stability at the CRM1’s binding site.

## Introduction

Active transport between the nucleus and cytoplasm is an essential regulatory mechanism for many cellular proteins. As a major nuclear exporter factor, chromosome maintenance protein 1 (CRM1; or exportin-1, XPO1) mediates nuclear export of hundreds of distinct cargo proteins by recognizing short sequence motifs called Nuclear Export Signal (NES)^1–3^. CRM1 shuttles between the nucleus and the cytoplasm, binds cargo molecules at high RanGTP levels inside the nucleus, traverses nuclear pore complex (NPC) as ternary cargo–CRM1–RanGTP complexes, and releases cargo into the cytoplasm upon hydrolysis of the Ran-bound GTP^4^. Since spatial re-localization of oncoproteins and tumor suppressor proteins is important in cancer cells, understanding of the NES can help the basic research about this process and can also help the discovery of anticancer agents^5^.

Classical NES motifs in the early studies were referred to as a cluster of hydrophobic residues, mostly leucines (hence also called Leu-rich NES), within a 10–15 residue-long sequence motif^1,6,7^. Many years of research on various export cargoes and randomization-and-selection screens showed that more residue types, such as Ile, Val, Met, and Phe, are also allowed at the hydrophobic positions of the CRM1-dependent NES signals^8,9^. These hydrophobic residues (Φ) are spaced with various patterns following the consensus Φ1-(x)_2–3_-Φ2-(x)_2–3_-Φ3-x-Φ4, where x denotes any amino acid. Later, structural studies of the CRM1 bound to NES peptides revealed another hydrophobic pocket in CRM1 that can bind to one more hydrophobic amino acid (Φ0)^10,11^. This site is less restricted to hydrophobic residues compared to others. Until recently, the existing 11 consensus patterns were defined by the peptide library-based study^9^ and structural analyses of CRM1-NES complexes^11–14^. They consist of four to five hydrophobic residues (Φ0-Φ4; generally, L, I, V, M, and F) which are bound to the corresponding hydrophobic pockets (P0-P4) in CRM1. Based on the pattern of these Φ’s and spacing sequences, the NES motifs are classified as class 1a, 1b, 1c, 1d, 2, 3, and 4. Additionally, compared to these classes, some peptides bind in the opposite (−) direction, making their Φ3-Φ4 positions bound to P0-P1 (class 1-reverse)^13^. Until recently, X-ray crystal structures of CRM1 bound to NES peptides of the 1a, 1b, 1c, 2, 3, 4, and 1a-reverse classes have been solved. Depending on the classes, the NES peptides showed distinct backbone conformations binding to the central portion of the hydrophobic groove of CRM1. One turn helix in the middle is remarkably conserved among all classes maintaining a hydrogen bonding with the Lys residue (Lys568) in human CRM1^14^.

Modeling short motifs or patterns like NES is a major research area in bioinformatics. Since NES motifs are essential regulators of the subcellular location of proteins in relation to cancer, cell cycle, cell differentiation and other important aspects of molecular biology, prediction of the NES motif is of great interest but still remains a challenge. Until now, more than 300 experimentally identified protein cargoes are recorded in databases such as validNESs^15^ and NESdb^16^ and over 1000 putative CRM1 cargoes were identified in a recent proteomics study^17^. Based on the ever-growing repertoire of the protein cargoes of CRM1, many attempts were tried to employ machine learning approaches to decide whether a given sequence has a CRM1-dependent NES motif or not. Several computational tools, such as NetNES^8^, NESsential^18^, NESmapper^19^, LocNES^20^, Wregex^21^, and NoLogo^22^ have been developed to predict NES motifs. Most of them are sequence-based predictors based on consensus pattern matching and calculation of biophysical properties such as disordered propensity, secondary structure components, and solvent accessibilities. To capture the diversity of the NES sequences, the consensus patterns were generally applied in the form of regular expression or position-specific scoring matrix (PSSM). Unfortunately, NES patterns are quite commonly observed in a large portion of the proteome so that the prediction based on these consensus patterns results in a high false positive rate. Since a functional NES needs to be solvent-exposed and not buried in a globular fold, Kırlı *et al*. applied these criteria and pattern matching to identify NES motifs in a set of validated, new CRM1 cargoes and found that functional NES motifs still could not be identified in a significant portion of them^17^. Moreover, sequences of functional NES motifs appear to be more diverse than previously appreciated. A large portion of experimentally defined NES regions does not match the current consensus patterns^17^. As a solution to reduce the high false positive rate, other biophysical features such as disorder propensity, secondary structure component, and evolutionary conservation were incorporated into machine learning algorithms like support vector machines (SVM) or neural networks^8,20^. However, the false positive rates remain high. In addition to the ever-expanding NES patterns resulting in many false positives when used in NES prediction, the limited information about direct CRM1 binding of the annotated NES regions is detrimental to develop accurate predictors using available data sets. Therefore, predicting NES motifs using only protein sequence information seems to have limitations, and the combination with structure-based predictions could be a new strategy to distinguish NES motifs and false positives.

In this study, using validated cargo protein sequences in NESdb and validNES, we provide a comprehensive look-up table which contains the location of the NES consensus patterns with the disorder propensity plots, conserved domain information, and the predicted secondary structure. This information could be useful for determining the most plausible NES region in the context of the whole protein sequence and for suggesting possibilities for some non-binders of the annotated NES regions. In addition, for the first time, we adopted the structure-based prediction of the NES sequences bound to the CRM1’s NES binding groove, using multiple crystal structures of CRM1-NES peptide as templates. For several experimentally validated NES peptides and false positive ones, we calculated the relative binding energy of the sequence segments at the CRM1’s binding pocket, and the prediction reliability of these binding energies was validated by the experimental binding affinities. Combining sequence-based and structure-based predictions, we suggest the novel and more straight-forward approach to identify NES sequences that bind directly to CRM1.

## Results and Discussion

### Deducing NES consensus pattern-matching sequences in candidate cargo proteins

**Figure 1:**
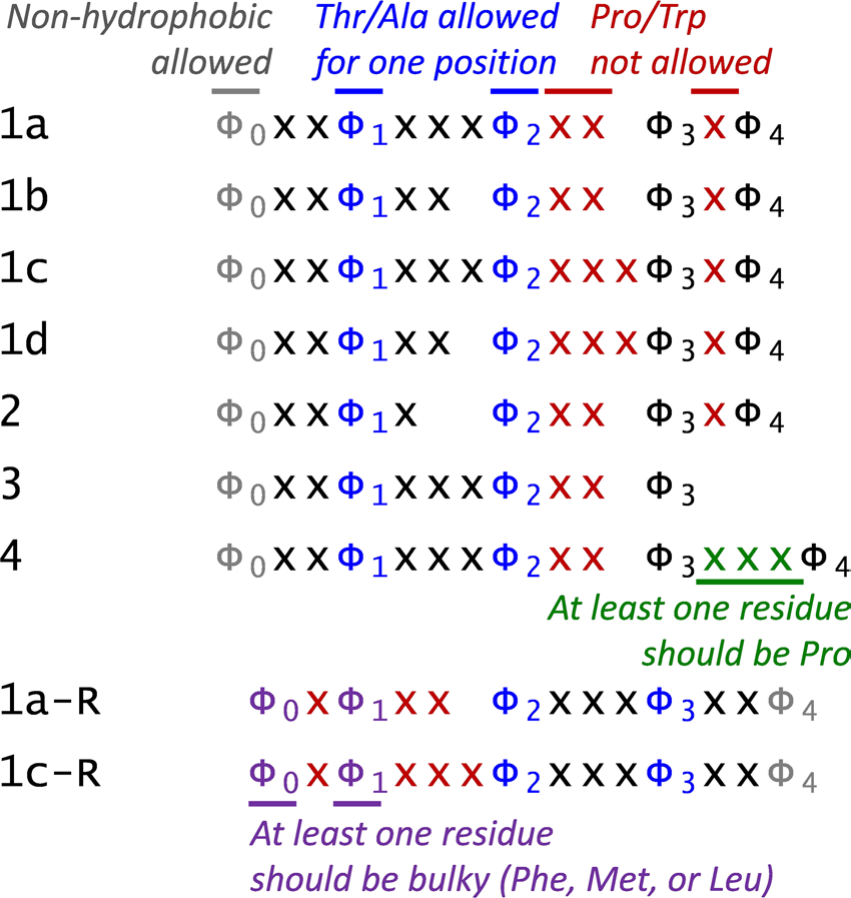
NES consensus patterns used in this study. For the hydrophobic positions, Φ_1–4_ are Leu, Ile, Val, Met, or Phe, and for the Φ_1_ and Φ_2_ positions, Thr or Ala is allowed for one position. Φ_0_ is not restricted to the hydrophobic amino acids. In the reverse classes, the criteria are applied in the opposite direction, and one of the Φ_0_ or Φ_1_ should be Leu, Phe, or Met. The spacer residues (x) can be any amino acid, but several positions have exceptions. The spacers in Φ_2_ [X]_n_Φ_3_XΦ_4_ (or Φ_0_XΦ_1_[X]_n_Φ_2_ in reverse classes) do not allow to have Pro or Trp. For class 4, at least one residue of the spacers in Φ_3_XXXΦ_4_ should be Pro to make a turn (as observed in the X-ray crystal structure of CRM1-X11L2 peptide).

Using the validated cargo protein sequences in NESdb and validNES (which have Leptomycin B (LMB)-sensitive data as evidence of CRM1-dependency), we extracted the NES consensus pattern-matching sequence segments based on the modified version of the Kosugi consensus^16,20^ as summarized in Fig. 1. All the possible consensus patterns are recorded and prioritized by the empirical class priority (see *Methods* for details). Based on these criteria, 4226 consensus-matching segments were extracted for 318 cargo protein sequences. Among them, 463 segments were treated as candidate NES motifs as they occur in regions that overlap to experimental evidence, and 3763 were treated as false positives (FPs). The experimental NES regions of 54 cargo proteins do not match the current consensus and are not considered in this study. Also excluded are four cargo proteins with no reported NES regions and five cargos with long reported NES regions (>25 residues) that do not have specific residues annotated. Among the consensus patterns, class 1a is the most abundant class (41%) as expected. Especially, compared to the false positive sequences, class 1a is observed more than twice as often in the candidate NES sequences. Classes 1c, 2, and 3 follow with 14~15%, class 1a-reverse is observed in 8.6%, and classes 1b, 1d, 4, or 1c-reverse seem to be quite rare (Fig. S1).

### A comprehensive look-up table of NES patterns in NES cargo proteins

**Figure 2:**
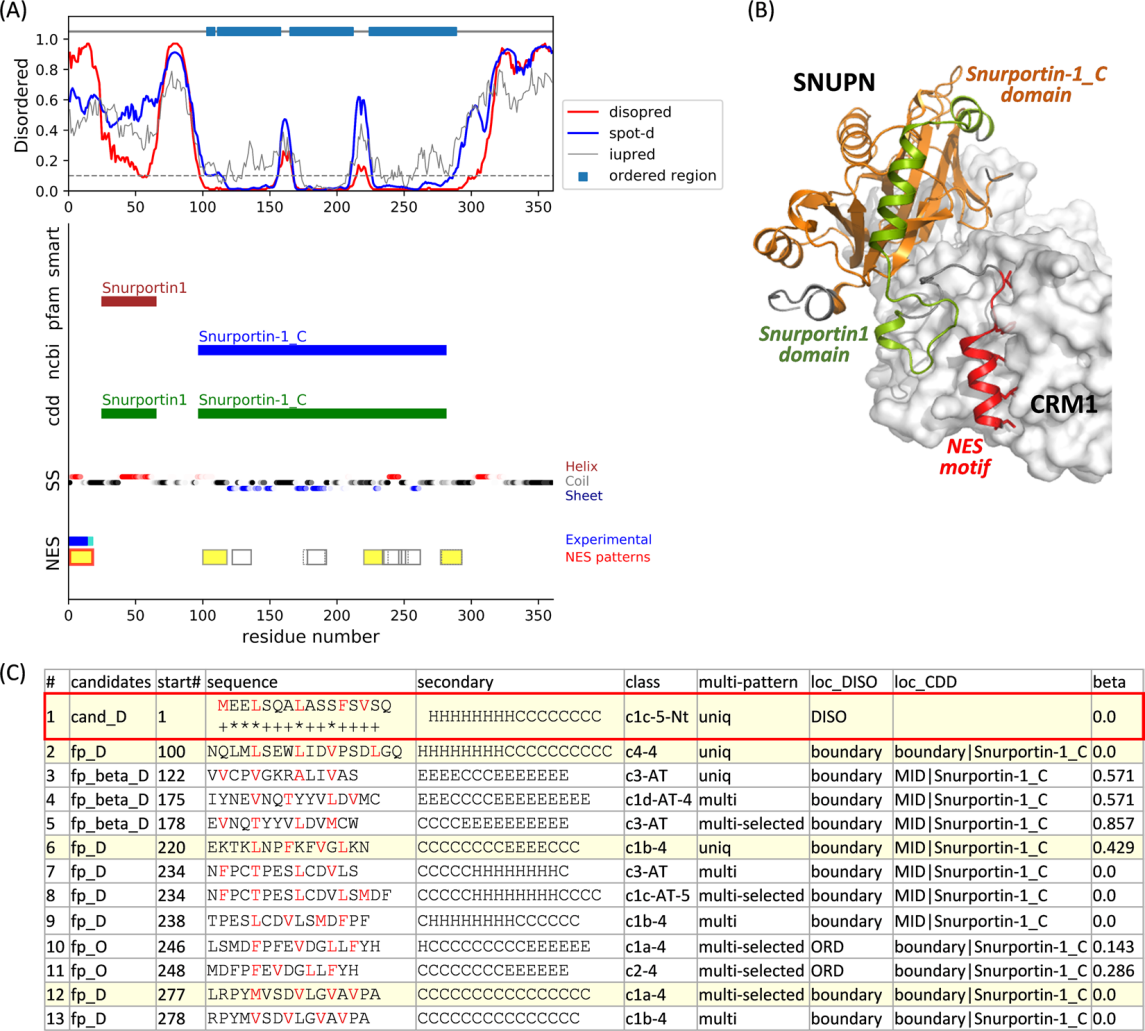
Location of the NES consensus patterns in Snurportin-1. (**A**) Disordered propensity, conserved domain information, predicted secondary structure, and the location of the consensus patterns are plotted together. The defined ordered region (by the cutoff value of 0.1; gray dashed line) is represented by the sky-blue box at the top. The regions of the conserved domains annotated in smart, Pfam, NCBI-curated, and CDD are marked in the middle. The predicted secondary structures (SS) were colored by red, black, and blue for α-helix, coil, and β-strand, respectively. The gradient of the color corresponds to the confidence level of the prediction. For the NES regions, experimentally validated regions are displayed in blue (with mutation data annotated in NESdb) and cyan (annotated as a functional sequence in NESdb or as a site in validNES). All the consensus pattern matching segments are located at the bottom. Segments not in the ordered regions and without β-strand predictions in the middle are highlighted in yellow. The red boxes are the pattern-matching segments overlapping with experimental evidence. (**B**) The crystal structure of CRM1-SNUPN complex structure (PDB id: 3GB8)^12^. SNUPN is displayed by the cartoon, and the validated NES motif, Snurportin1 domain, and Snurportin-1_C domain are colored in red, green, and orange, respectively. CRM1 is represented by a white surface. (**C**) The list of the pattern-matching sequences in SNUPN. In the ‘candidates’ column, NES candidates and false positives are annotated with “cand” and “fp,” respectively. If the segment is located in the disordered or boundary region, it is flagged with “_D” while in the ordered region, it is flagged with “_O.” If the segment’s β-strand content is over 0.5, it is flagged with “_beta.” In the ‘sequence’ column, hydrophobic positions are colored in red, and the positions with the experimental evidence are marked with ‘*’ (mutation) and ‘+’ (functional sequence in NESdb or sites in validNES). The values in ‘diso,’ ‘spotd,’ and ‘iup’ are the average disordered propensity for the segment calculated by DISOPRED3, SPOT-Disorder, and IUPRED2A, respectively. The locations with respect to disordered/ordered region or conserved domains are listed in the ‘loc_DISO’ and ‘loc_CDD’ columns. ‘beta’ is for the β-strand content in the middle of the segment.

In order to make the NES motif to be accessible to CRM1-binding, the motif should not be located in the compactly folded protein domains. The NES motif may be located at the N-terminus, at the C-terminus, or within an unstructured region of an export cargo^11^. Therefore, for a precise prediction of the export signals, it is crucial to consider the motifs’ location with respect to protein domains and disordered regions. For all possible NES consensus patterns of the cargo proteins that we extracted, we analyzed the relationship with the protein ordered/disordered regions, known domains, and their predicted secondary structures, and provide a comprehensive online table. For a given full protein sequence, we plotted the disordered propensity, the location of the known domains, the predicted secondary structures, and all possible NES consensus regions (Fig. 2). For a given entry, the information annotated in NESdb or validNES, such as evidence of CRM1-dependency, mutation data, functional sequences or sites, is listed together. The locations of all NES consensus-matching segments are marked together with the experimentally validated regions (Fig. 2A, the bottom of the plot). The reference databases (NESdb, validNES, and UniProt), protein visualization tool (ProViz)^23^ and the structure and model database (SWISS-MODEL repository)^24^ are linked for user convenience, and the filter for easy look-up is also provided. This table could be useful for determining the most likely NES region in the context of a whole protein sequence. The online table is accessible via: http://prodata.swmed.edu/nes_pattern_location/.

### NES candidates in the disordered or ordered regions

Even if a sequence motif can be fitted to the NES consensus, a motif that is located deep in the globular fold can hardly bind to CRM1 unless the region unfolds. In some cases, it may be possible to unfold and bind, but we assume that these cases would be very limited. Also, short linear interaction motifs like NES motifs have been proposed to be locally disordered to facilitate dynamic interactions with their binding partners, and the NES prediction algorithms have used disorder context to help distinguish correct NES motifs from false predictions^18,20^. However, NES motifs do not necessarily have to locate in the fully disordered region. Indeed, we have observed that some NES candidates are located in the fully disordered regions, but others are located next to ordered or “boundary” regions. Therefore, we employed the disorder propensity as a pre-filter to remove the segments located in the “highly” ordered regions.

Various computational tools have been developed for analyzing potential intrinsic disorder of protein sequences and were quite successful owing to clear association between disordered propensity and sequence features such as low complexity or high aromatic composition. We utilized DISOPRED3^25^ and SPOT-disorder^26^, which use homologous sequences’ alignment-based profiles for detecting disordered regions, and IUPred2A^27^ which is much faster since it does not rely on the sequence alignment. Disordered regions for some proteins are quite differently predicted depending on the programs. In order to define ordered and buried regions with high confidence, we applied strict cutoff values (~0.1) to decide the order/disorder border lines (note that the most of the programs’ cutoff value for disordered regions are ~0.5). If a residue’s disorder propensities predicted by both DISOPRED3 and SPOT-Disorder are below 0.1, the residue is defined as in highly ordered region (note that the predicted values by IUPred2A are also recorded for the reference).

**Figure 3:**
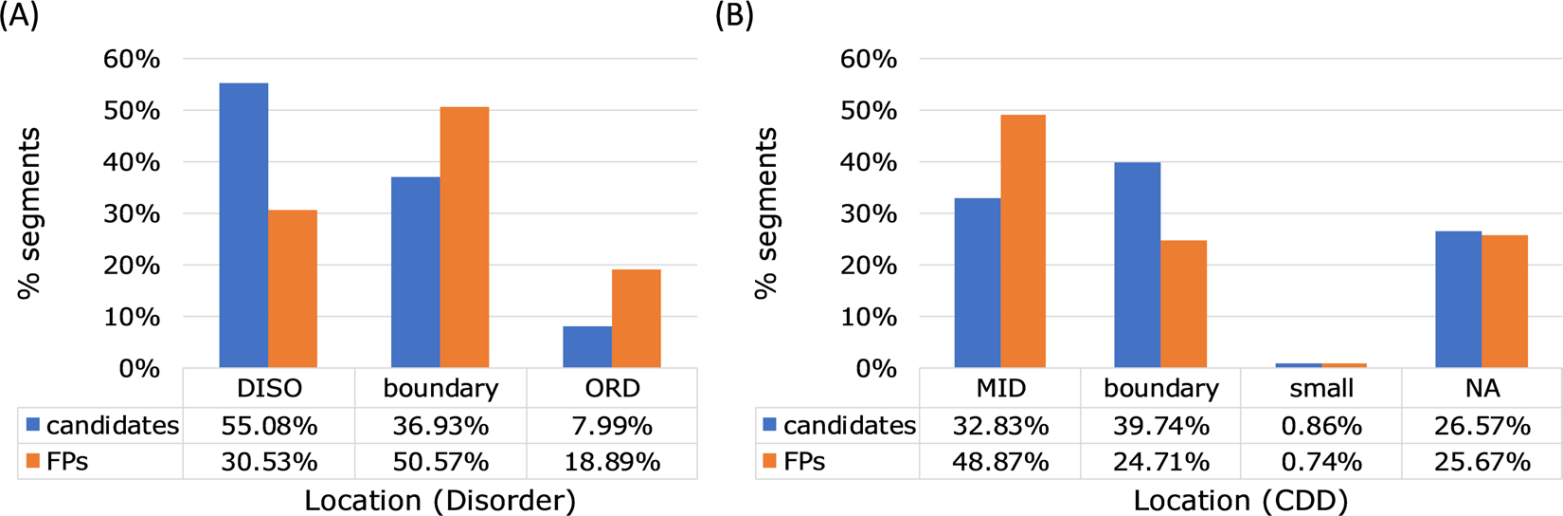
Location of the candidate NES and false positive sequences. (**A**) Location with respect to the disordered or ordered regions. DISO: located in the disordered region; boundary: located at the end of the highly ordered region; ORD: located in the highly ordered region. (**B**) Location with respect to the known domains annotated in CDD. MID: located in the middle of the domain; boundary: located at the end of the domain; small: located in the small domain (<50 residues); NA: located in the region with no annotated information.

**Figure 4:**
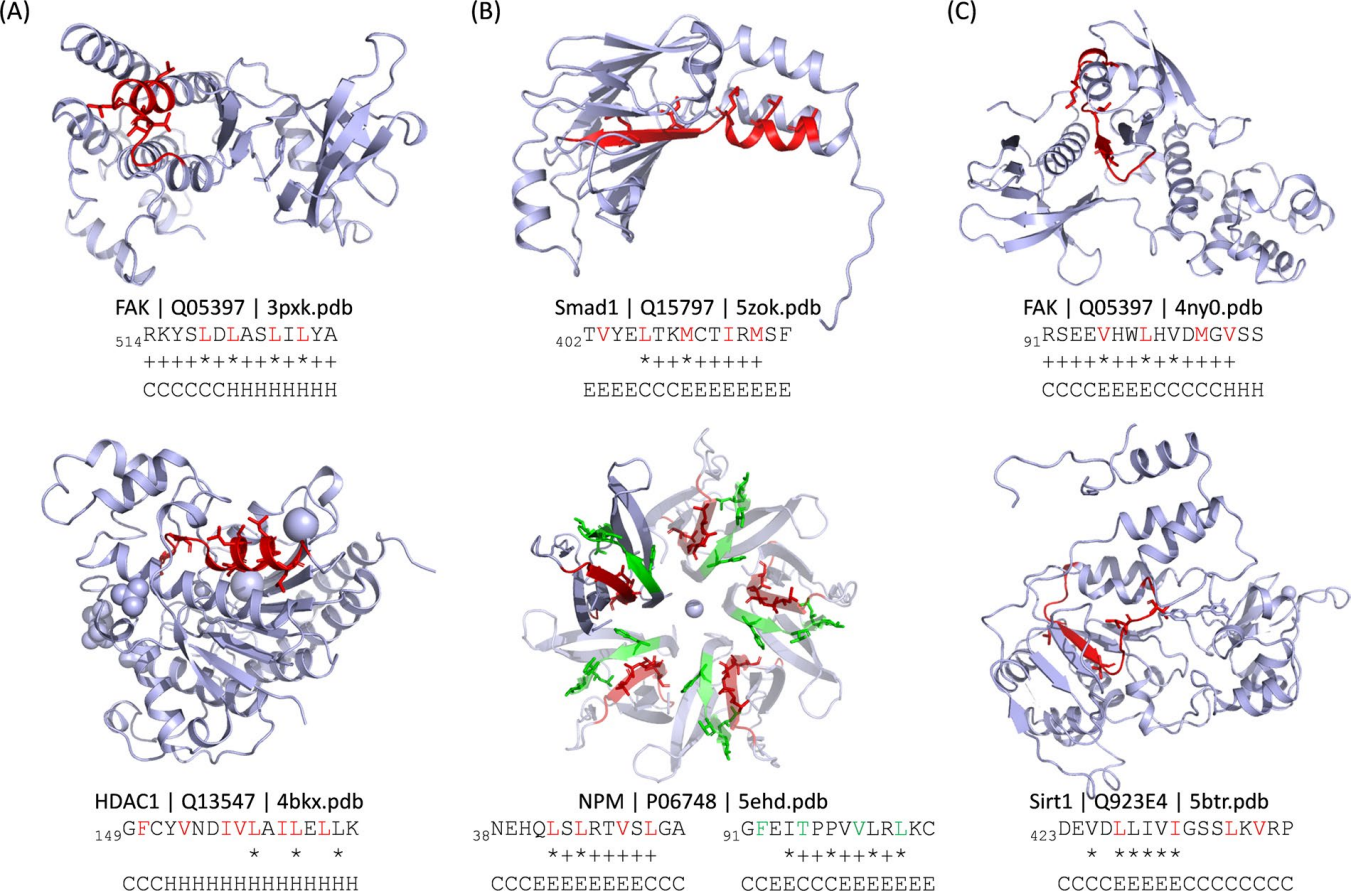
Examples of possible non-binders to CRM1. (**A**) Segments located in the ordered globular fold. (**B**) Segments with β-strands in the middle. (**C**) Segments located in the ordered region and have β-strands in the middle. The hydrophobic residues are colored in red or green in the sequences and displayed as sticks in the structures.

As shown in Fig. 3A, 55% of the NES candidate motifs are located in the disordered region, and 37% are found in the boundary region between the ordered and disordered parts. Only 8% of the NES candidate motifs are located in the highly ordered region. Among the 361 candidate motifs, 37 segments (for 20 cargo proteins) are located in the highly ordered region which may have less possibility to be accessible to CRM1 binding. For example, HDAC1 (uniport ID: Q13547) has a reported NES motif with a mutation data (L158A/L161A/L164A) for nuclear export^28^. This region can be fitted to the classes 1c, 2, or 3, but it is located in the highly ordered region. The crystal structure of HDAC1 (PDB ID: 4bkx) showed that this segment is buried in the globular domain and seems unlikely to be accessed by CRM1 (Fig. 4A). Note that in case of its homolog HDAC5, the candidate NES motif (_1081_EEAE**T**VSA**M**AL**L**S**V**GA_1096_, class 1a) is located in the disordered region after the conserved Hist_deacetyl domain and found to directly bind to CRM1. The similar region (after the Hist_deacetyl domain) in HDAC1 (_358_Y**L**EK**I**KQR**L**FEN**L**R**M**LP_374_, class 1c) could be also considered as a possible NES motif of HDAC1. Table S1 lists the NES candidate motifs located in the highly ordered region and Fig. 4A,C shows some examples for these segments in the available 3D structures.

In case of the false positives, the segments located in the highly ordered region is 19%, a larger percentage than those of the candidate NES motifs (note that the segments in the ordered region are far lower than those in the disordered region since we use the stringent cutoff for defining ordered region). The false positives in the disordered or boundary regions are 31% and 51%, respectively.

### CDD domains and NES locations

To analyze the candidate NES motifs’ location with respect to the conserved regions, we extracted the conserved domain information for the cargo protein sequences using the four different databases, i.e., SMART, Pfam, NCBI-curated, and Conserved Domain Database (CDD). As shown in Fig. 3B, only 33% of the candidate NES regions are located in the middle of the CDD domains, and 40% is in the boundary region. It seems that the NES regions do not necessarily locate in the protein domains. Rather, the known domains are often considered to form folding units, masking the possible motifs from binding other proteins. In case of the false positives, more than half are located in the middle of the known domains. It may be because the hydrophobic residues are commonly located in the protein core or domains.

### Secondary structure components of the NES peptides

Crystal structures of CRM1-bound NES peptides have been resolved for the classes 1a, 1a-reverse, 1b, 1c, 2, 3, and 4. They showed distinct backbone conformations that match their hydrophobic positions to the corresponding hydrophobic pockets in CRM1. Structural analysis, as well as secondary structure prediction of NES motifs, suggest that most NES motifs contain α-helices or helix-to-extended conformation^12–14^. The class 1d is also expected to have helix-strand, and other reverse (−) classes are likely the reverse of their (+) counterparts^14^. The common feature of the backbone conformations among the classes is one turn of helix at the region from Φ2 to Φ3^14^.

In our analysis of the 361 candidate motifs, 36 segments (for 23 cargoes) have a β-strand conformation in the middle (β-strand contents of the middle part is >50%) (Table S2). Among them, 11 segments were confirmed to have β-strands in the available X-ray or solution structures. For example, NPM has two reported NES regions, but both of them are predicted to form β-strands in the middle of the segments. As shown in Fig. 4B, the two segments are both β-strands located in the middle of the jelly-roll fold. Indeed, both regions were also reported to be quite weak binders of CRM1^29^ and the sequence of 42–61 failed to bind CRM1 in GST-pulldown assay (Chook Lab, unpublished results; annotated in NESdb). The candidate NES region in TDP-43 is also located in β-strands within a folded globular RRM domain, and it is recently validated to be a non-binder to CRM1 rather it is exported by passive diffusion^30^. For six segments, there is no experimentally determined structure, but homology models showed the β-strands for the segments. For 17 segments, no structural information is available. For two segments, the conformation in the modeled structures (with sequence identities of 79% and 98%, respectively) are found to be helix reflecting the limitation of the secondary structure prediction.

### Evaluation of the stability of the NES peptides at the CRM1 binding groove based on structure modeling

Recent structural works of CRM1 complexed with various cargo sequences expand the possible consensus patterns^13,14^. Also, the NES-binding site in RanGTP-bound CRM1 is found to be quite rigid, and the peptides display CRM1-dependent NES activity only if their backbone conformations can place a sufficient number of the hydrophobic residues into the CRM1’s binding groove^11^. The adapting conformation of the peptides can be efficiently analyzed by structure-based modeling methods so that the application of the structural information can advance more accurate NES prediction.

**Figure 5:**
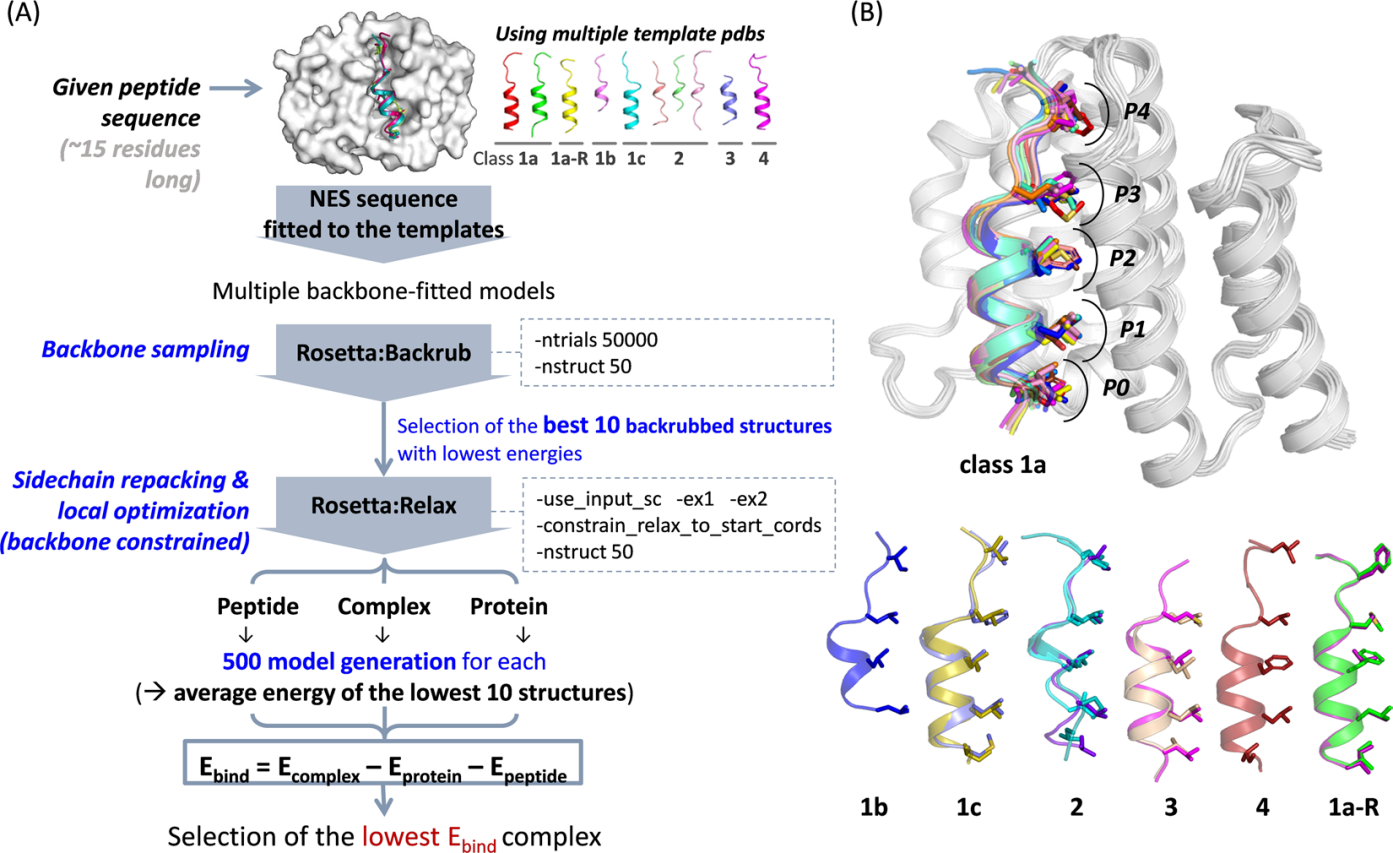
Structure-based prediction of the stability of CRM1-NES peptide complex. (**A**) CRM1-NES peptide complex model generation and E_bind_ calculation procedure. (**B**) Generated models for the complex structures of CRM1-NES peptides with lowest E_bind_. Class 1a peptides are displayed with CRM1 (in white) at the top. The hydrophobic (Φ) residues of these NES peptides (shown in the sticks) occupy the corresponding hydrophobic pockets (P0-P4) in CRM1. Peptides of other classes are shown at the bottom with the hydrophobic residues shown in the sticks.

Using the reported NES peptides with experimental binding affinities^14,31^ as a benchmarking set (Table 1), we evaluated the binding energy (E_bind_) for a given peptide sequence at the CRM1 groove (see *Methods* for details). Binding energy can be assumed as relative stability of the protein(CRM1)-peptide(NES) complex structure compared to the protein itself and free peptide. The lower the binding energy, the higher the possibility for the peptide segments to bind at CRM1. Multiple crystal structures of CRM1-NES peptide (super PKI and MVM-NS2 for classes 1a; FMRP-1b for class 1b; SNUPN for class 1c; FMRP and SMAD4 for class 2; HIV-Rev for class2-rev type; X11L2 for class 4; and CPEB4 for class 1a-reverse; class 1a templates can be used to fit class 3 NES peptides) were utilized as templates. The model generation and energy calculation process are summarized in Fig. 5A.

**Table 1:** Peptide sequences of the validated NES motifs or false positives used in the structure-based modeling.

Protein	Class	NES sequence	K_D_ (nM)^§^	ref.
MVM NS2	1a	_77_ST**V**DE**M**TKK**F**GT**L**T**I**HD_93_	2	^31^
^*^super PKI	1a	_34_N**L**NE**L**ALK**L**AG**L**D**I**NK_49_	4	^31^
PKI	1a	_34_NSNE**L**ALK**L**AG**L**D**I**NK_49_	34	^31^
ADAR1	1a	_121_RG**V**DC**L**SSH**F**QE**L**S**I**YQ_137_	69	^31^
MEK1	1a	_28_TN**L**EA**L**QKK**L**EE**L**E**L**DE_44_	70	^31^
Pax	1a	_264_RE**L**DE**L**MAS**L**SD**F**K**F**MA_280_	700	^31^
^*^CPEB4-R	1a	_395_RM**I**DI**L**SSE**L**SH**M**D**F**TR_379_	710	^31^
NPMmutA	1a	_278_MTDQEA**I**QD**L**CLA**V**EE**V**S**L**RK_298_	790	^31^
HDAC5	1a	_1081_EAE**T**VSA**M**AL**L**S**V**G_1095_	1600	^31^
p73	1a	_364_NFEI**L**MK**L**KES**L**EL**M**E**L**VP_382_	2000	^31^
^*^hRio2-R	1a	_405_GK**I**EE**L**AQN**F**ET**M**E**F**SR_389_	2600	^31^
Stradα	1a	_413_G**I**FG**L**VTN**L**EE**L**E**V**D_427_	10300	^31^
^*^FMRP-1b	1b	YLKE**V**DQ**L**RA**L**ER**L**Q**I**D	3000	^14^
SNUPN	1c	_1_**M**EE**L**SQA**L**ASS**F**S**V**SQDLNS_20_	12500	^31^
HPV E7	1c	_73_HVD**I**RT**L**EDL**L**MGT**L**G**I**VC_91_	34000	^31^
HIV Rev	2	_73_LQ**LP**P**L**ER**L**T**L**DC_85_	1180	^31^
FMRP	2	_424_LKE**V**DQ**L**R**L**ER**L**Q**I**D_438_	2000	^31^
SMAD4	2	_134_ERVVSPG**I**D**L**SG**L**T**L**Q_149_	4600	^31^
mDia2	3	_1157_SVPE**V**EA**L**LAR**L**RA**L**_1171_	1600	^31^
CDC7	3	_456_QD**L**RK**L**CER**L**RG**M**DSSTP_473_	20000	^31^
X11L2	4	_55_SS**L**QE**L**VQQ**F**EA**L**PGD**L**V_72_	1500	^31^
CPEB4	1a-R	_379_RT**F**D**M**HS**L**ESS**L**ID**I**MR_395_	800	^31^
hRio2	1a-R	_389_RS**F**E**M**TE**F**NQA**L**EE**I**KG_405_	2800	^31^
^*^PKImut1 (I47A)	—	_34_NSNE**L**ALK**L**AG**L**DANK_49_	150000	^31^
^*^PKImut2 (L42A/L45A)	—	_34_NSNE**L**ALKAAGAD**I**NK_49_	900000	^31^
^†^APC	1a	_163_AQ**L**QN**L**TKR**I**DS**L**P**L**_174_	(−)	^33^
^‡^Cyclin D1	1a	_281_VD**L**AC**T**PTD**V**RD**V**D**I**_295_	(−)	—
APRIL	1b	_106_LEP**L**KK**L**EC**L**KS**L**D**L**_120_	(−)	^33^
^‡^hTERT	1c	_965_KAGRN**M**RRK**L**FGV**L**R**L**KC_982_	(−)	—
DcpS	1c	_136_TEKH**L**QKY**L**RQD**L**R**L**_150_	(−)	^33^
Cdk5	2	_133_LINRNGE**L**K**L**AD**F**G**L**_147_	(−)	^33^
^†^FGF1	2	_138_THYGQKA**I**L**F**LP**L**P**V**_152_	(−)	^33^
COMMD1	3	_171_ILKT**L**SE**V**EES**I**ST**L**_185_	(−)	^20^
DEAF1	1a-R	_452_SW**L**Y**L**EE**M**VNS**L**LNTAQQ_469_	(−)	^13^
SGN5	1a-R	_221_YA**L**E**V**SY**F**KSS**L**DRKLL_238_	(−)	^13^
^†^COMMD1–2	1a-R	_164_DE**V**K**V**NQ**I**LKT**L**SE**V**EES_181_	(−)	^13^
^†^ELF3	1a-R	_111_R**L**V**F**GP**L**GDQ**L**HAQLR_126_	(−)	^13^

^*^Engineered or mutated (underscored residues are the ones inserted or mutated).

^†^Do not fit the consensus in Fig. 1 (due to Pro or do not have a bulky residue at Φ3/4 in the class 1a-R).

^‡^Unpublished data.

^**§**^(−) means no binding determined by pull-down binding assay.

**Figure 6:**
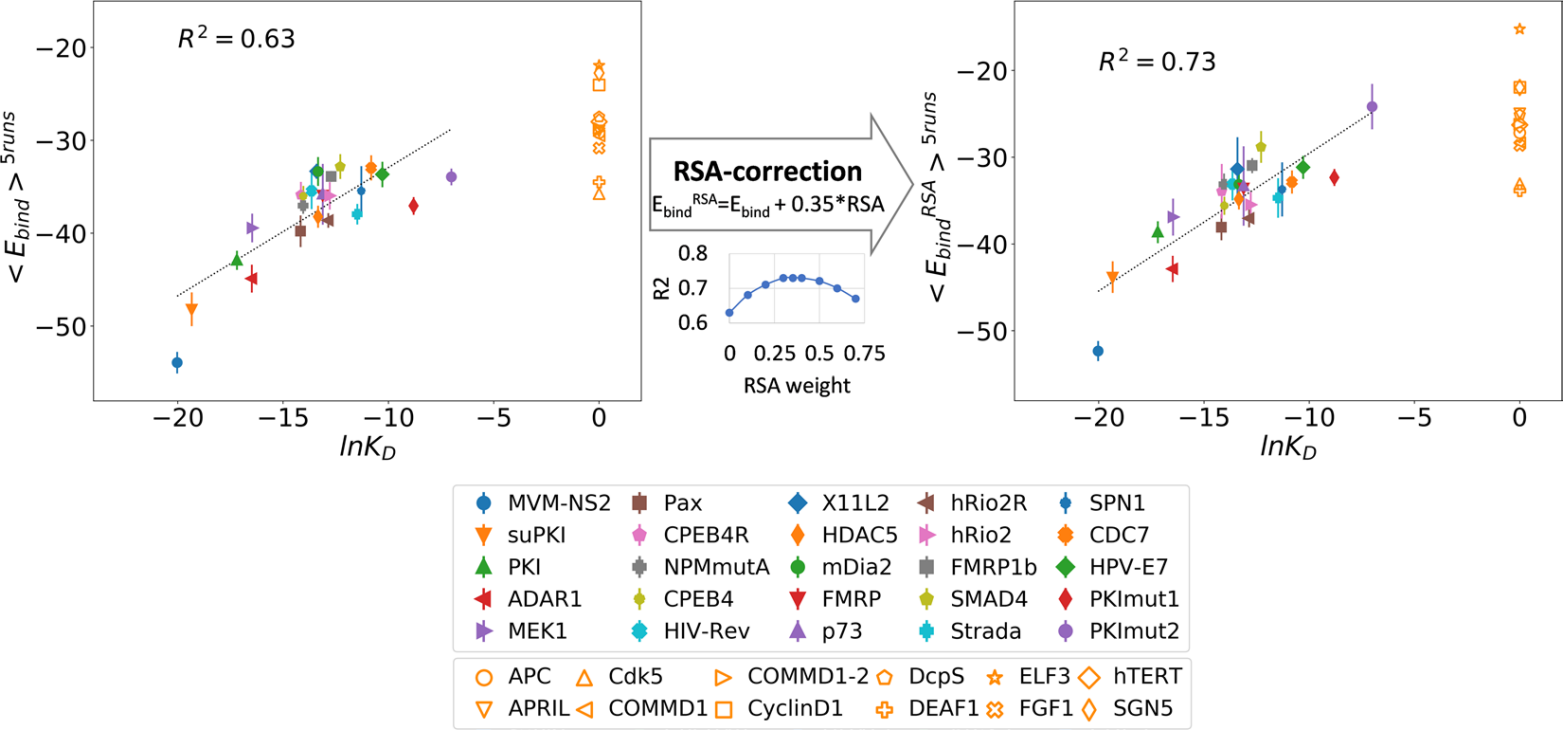
Correlation between the binding energies and the experimental K_D_ values. The binding scores are averaged in the five independent runs (<E_bind_>^5runs^; <E_bind_^RSA^ >^5runs^ for the RSA-corrected values) and compared to the logarithm of K_D_ values (lnK_D_). The CRM1-binders with K_D_ values are shown in filled markers with error bars which are the standard deviation during the five runs. The false positives are shown in orange empty markers. In the middle, the correlation between R^2^ and the weights for RSA during the E_bind_ correction is shown. The weight of 0.35 were applied for calculation of E_bind_^RSA^.

Final model structures showed that all classes were predicted well with their Φ residues bound to the corresponding hydrophobic pockets (Fig. 5B). The calculated E_bind_ selected the right template for each class, and it can be utilized to find the most plausible class when multiple consensus patterns are found in one segment. The calculated E_bind_ values correlated quite well to the experimental K_D_ values (Fig. 6, left; *R*^2^~0.63; *Pearson’s r*~0.79 with *p* = 2e − 6). However, in the case of the two PKI mutant peptides which have extremely low binding affinities, the E_bind_ scores are not quite distinguishable from those of the weak binders such as SNUPN, SMAD4, and HPV-E7. In case of the PKI double mutant peptides, we found a large interface cavity at the binding interface with CRM1 (Fig. S3A), but this feature, definitely detrimental to binding, is not well reflected in the modeling process or energy calculation. To penalize the interface cavity of the complex structure, residue solvent accessibility (RSA) for key interface residues (Fig. S3B) is calculated using the NACCESS program^32^ and treated as another scoring term. The RSA-corrected E_bind_ scores (E_bind_^RSA^) is obtained by calculating E_bind_^RSA^ = E_bind_ + *w*∙RSA (*w* is the weight for the RSA term and is optimized to maximize the correlation) (Fig. 6, middle). E_bind_^RSA^ gave improved correlation (Fig. 6, right; *R*^2^~0.73; *Pearson’s r*~0.86 with *p* = 5e-8).

**Figure 7:**
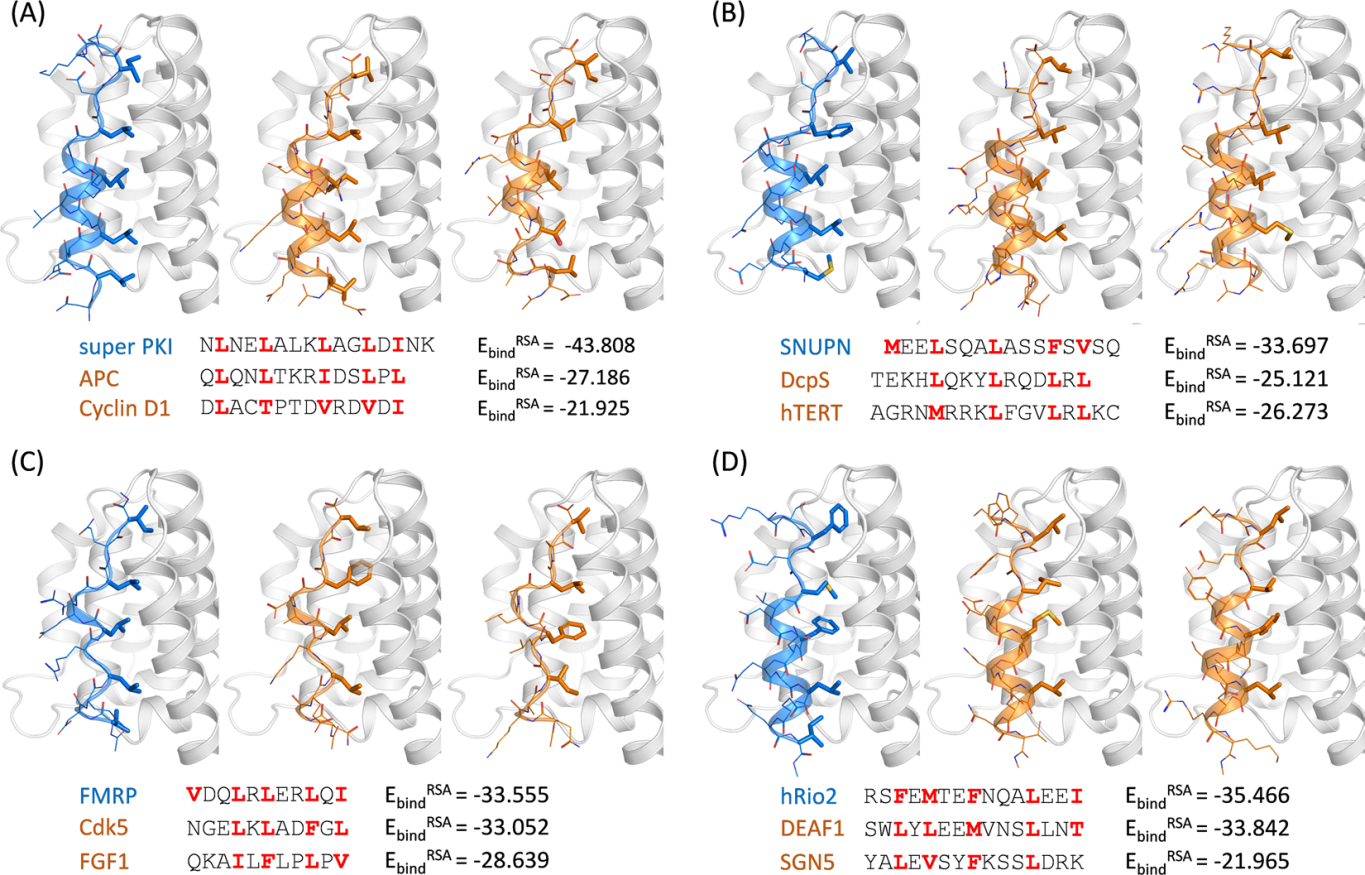
Comparison of the structural models and binding energies of the CRM1-binding NES motifs (blue) and false positive sequences (orange). CRM1 structure is colored in white. The hydrophobic residues are colored in red in the sequences and displayed as sticks in the structures. The spacer residues are represented as lines.

For comparison, several false positive sequences that can be fitted to NES consensus but are experimentally validated as non-binders (determined by pull-down binding assay)^13,33^ are subjected to modeling with the same procedure. Interestingly, these false positives showed significantly higher E_bind_ scores reflecting their low binding affinities at the CRM1 binding groove. Notably, the peptides such as COMMD1 (_164_DE**V**K**V**NQ**I**LKT**L**SE**V**EES_181_) and ELF3 (_111_R**L**V**F**GP**L**GDQ**L**HAQLR_126_) were not fitted to the right template (i.e., the lowest E_bind_ complex is not the class 1a-R structure). It suggests that these sequences could be energetically unstable when their backbone conformations are fitted their hydrophobic residues to CRM1 hydrophobic pockets. In case of the false positive peptides fitted to the right template (Fig. 7), the backbone conformation and the Φ residues may appear to be pretty similar to the true positive ones; however, they showed inferior binding energies. In some cases, such as Cyclin D1 (Fig. 7A, middle) or FGF1 (Fig. 7C, right), the backbone conformation seems to be not maintained well when presenting the Φ side chains into the pockets.

We expect the merit of this structure-based, energy-based method is to discriminate true positive and false positive with similar sequence patterns, by analyzing energetic differences at the CRM1 binding site via full-atom modeling. This atomic-level energetic analysis cannot be deduced by using the only sequence. In this perspective, our method would suggest novel approaches to find the CRM1-binding NES motifs. We cannot ignore the fact that the interaction between CRM1 and a whole cargo protein can be more than that of the CRM1-NES peptide^10^; however, it is extremely difficult to consider extra contacts between CRM1 and cargo’s whole structure which may be different depending on each cargo. Based on our previous result describing the strength of the CRM1-NES peptide interaction correlated to the nuclear export activity^31^, we assume that the energy prediction between CRM1 and NES peptide is a practical strategy.

For evaluating the performance, we compared our results to those of other sequence-based methods, i.e., NetNES^8^, NESmapper^19^, and LocNES^20^ (Figs S4–S20). Using the whole sequences of 17 proteins in Table 1, we extracted 19 positive cases (regions annotated as NES motifs in the NESdb or validNES database with mutational evidence) and 341 negative cases (non-NES regions with consensus pattern-matching). As shown in Table S3, E_bind_ score performs the same as LocNES in terms of recall rate (both predicts 17 true positives out of 19 experimentally verified NES cases). On the other hand, E_bind_ outperforms LocNES in terms of specificity and false positive rate. E_bind_ recorded 23 cases of false positives while LocNES predicted nearly the double amount of false positives (40 cases). NetNES showed better specificity (true negative rate (TNR): 0.988) than our method (TNR: 0.933). However, its recall rate (sensitivity or true positive rate (TPR): 0.474) was much lower than our method (TPR: 0.895). Our method seems to work well enough compared to these available methods. It effectively decreases false positives while maintaining a high recall rate, showing the best performance with respect to the balance of precision & recall (F_1_ score), and effectiveness (DOR).

### Possibility of non-binders to CRM1 among the NES-annotated regions

The databases like validNESs^15^ and NESdb^16^ provide valuable information on NES research, however, defining CRM1-dependent NES regions is still a difficult task. The expanding NES patterns result in many false positives. Also, the lack of information showing direct CRM1 binding to many annotated NES regions prevents development of accurate predictors using available data sets. Most published experimental studies were focused on showing that a protein is an export cargo, by deletion of the whole region encompassing a candidate NES or by mutation of all the suspected hydrophobic residue positions. These perturbations are drastic and may affect structural stability and result in defects of functions other than CRM1-binding and nuclear export. Therefore, one should interpret the experimental data carefully to identify the CRM1-binding NES location, and it is always possible that regions which have been annotated as experimentally validated are not in fact functional NES motifs. Indeed, some of the annotated NES regions were found in the buried (highly ordered) protein domains (Fig. 4A,C). Some others can form β-strands in the middle of the segment (Fig. 4B,C) which would be rare in real NES sequences. Candidate segments that form β-strands and are located in the ordered region are observed in three cargoes including FAK (_91_RSEE**V**HW**L**HVD**M**G**V**SS_106_), MoKA (_190_K**I**QT**L**H**L**VG**V**N**V**PE_203_), and Sirt1 (_423_DEVD**L**LIV**I**GSS**L**K**V**RP_239_). We suggest that these segments have high possibility to be non-binders to CRM1 unless they unfold or transform their conformations upon specific conditions. Some cargo proteins might be exported following other events such as binding to an NES-containing adaptor protein.

**Figure 8:**
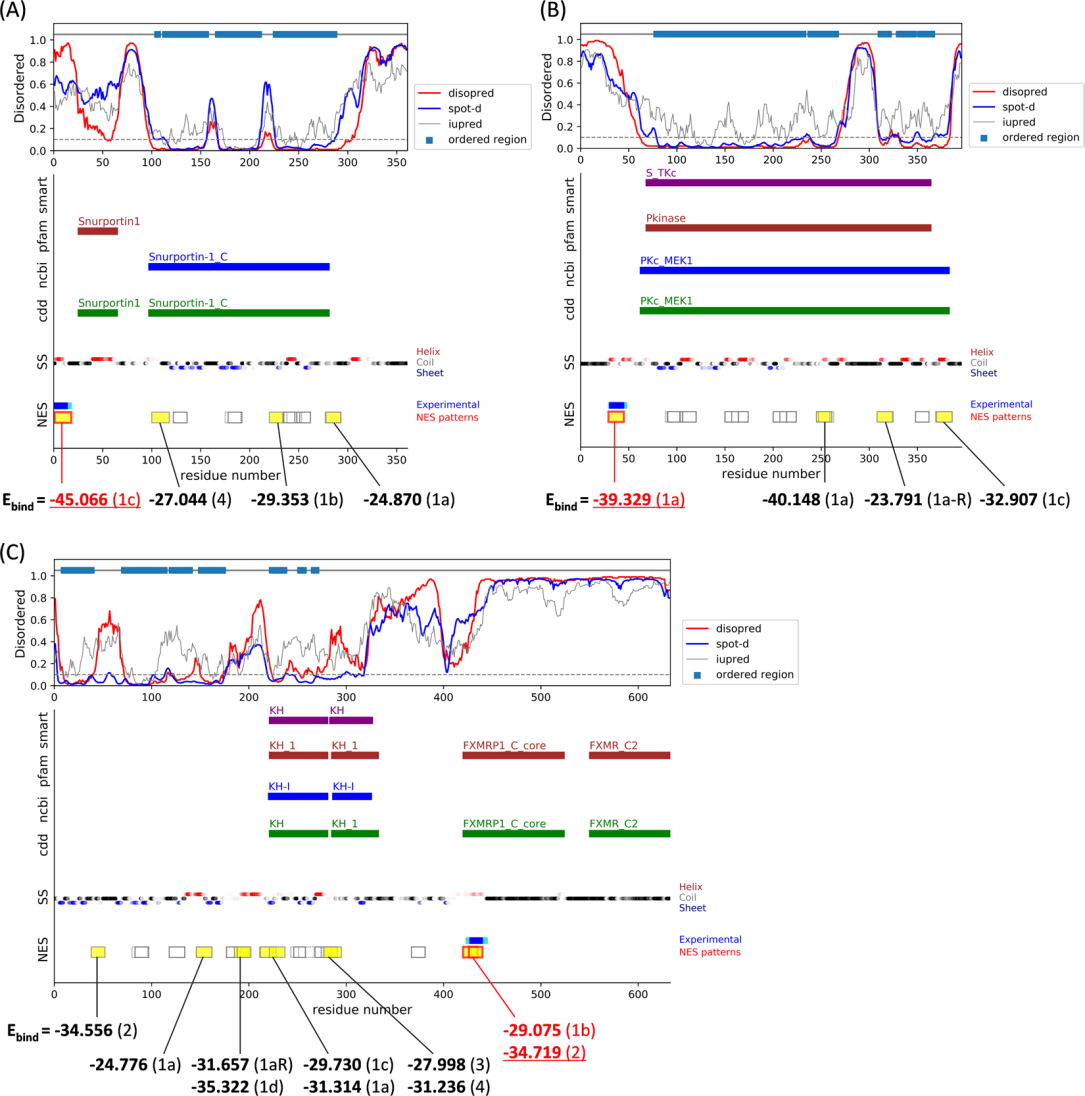
Distinguishing CRM1-binding NES motifs and false positives by E_bind_. Location of NES consensus and their binding energies in (**A**) Snurportin-1 (O95149), (**B**) MEK1 (Q05116) and (**C**) FMRP (Q06787). The description for the plots is same as Fig. 2. The calculated E_bind_ scores for the important segments (pattern-matching segments which are not located in the highly ordered region and do not have β-strand conformation in the middle; yellow highlighted) were displayed together. The E_bind_ scores of the candidate NES motifs were underlined and marked in red. The classes of the consensus patterns are marked in parentheses.

Even if a segment fits the NES consensus and also satisfies the location criteria, these criteria are still not enough to locate the real NES segments in the whole protein sequence (see yellow highlighted segments in the online table). We tested the E_bind_ calculation to the all possible segments of the natural cargo proteins listed in Table 1. If a segment cannot form an energetically stable complex at the CRM1’s NES binding groove, it is likely a non-binder to CRM1. As shown in Fig. 8, the NES candidates are likely to have the lower E_bind_ scores compared to other false positive segments. Among the seventeen cases, eleven cases have the NES candidate motifs with the lowest E_bind_, and four cases have the NES regions with the second lowest E_bind_ but the difference between the lowest and second lowest is usually marginal (less than 2). Although the data set used in the structure-based modeling is quite small, the resulting binding energy values can discriminate between CRM1 binders and false positives. This structure-based prediction method can be utilized as one of the features to find real CRM1-dependent NES peptides in the pool of numerous false positive sequences.

## Conclusion

In summary, we analyzed the structural prerequisites for CRM1-dependent NES motifs, i.e., accessibility (by locating disordered/ordered regions), adapting conformation (by predicting secondary structures), and the stability at the binding site (by applying structure-based modeling to calculate binding energies). The comprehensive table including all the possible consensus patterns with the disordered propensity plot, conserved domain information, and the predicted secondary structures provide valuable information for determining or correcting the most probable NES regions.

In light of the currently resolved crystal structures of CRM1-NES peptides with diverse classes, we modeled the CRM1-NES peptide complex structures and calculated the stability of the NES peptides at the CRM1 binding groove. The resulting binding energies correlate well to the experimental binding affinities, and we can distinguish the real NES motifs and false positives which both match NES consensus patterns. Also, we do not rely on the input sequence’s pattern, rather use the energy function to select the most energetically favorable class template. Therefore, if the multiple patterns exist in one peptide segment, this energy calculation can be a tool to predict the peptide’s conformation when it binds to CRM1. Although the method can still be improved, this study provides a starting point to predict NES motifs by combining sequence-based and structure-based approaches. Because our method is template-based modeling, it is difficult to adequately model NES motifs of classes other than those of the templates. Since newly discovered NES motifs often deviate from the established consensus patterns, more structural information is definitely needed not only to understand new consensus patterns and NES-CRM1 binding mechanism but also to more accurately predict NES motifs.

## Methods

### Extraction of the NES consensus sequences

For the cargo proteins which have LMB sensitive data as CRM1-dependency annotated in NESdb^16^ and validNES^15^, the NES consensus-matching sequence segments were extracted by utilizing the modified version of the Kosugi consensus^16,20^ (Fig. 2): Φ1-X_1,2,3_-Φ2-[^PW]_2_-Φ3-[^PW]-Φ4; Φ1-X_2,3_-Φ2-[^PW]_3_-Φ3-[^PW]-Φ4; or Φ1-X_2_-Φ2-X[^PW]_2_-Φ3-[^PW]_2_-Φ4 ([^PW] is any of the 20 amino acids except Pro and Trp; Ala or Thr can be used only once at Φ1 or Φ2; X stands for any amino acid). If one segment or segments in the similar region (difference between the two segments’ starting residue numbers <5) can be fitted to multiple patterns, all the possible patterns are recorded but prioritized based on the fact that: (i) the class 1a pattern is the most frequently observed class in the validated NES sets, suggesting that it interacts more preferentially with CRM1 than other classes^9,16,22^; (ii) in the current NES databases, class 3 sequences are as prevalent as NES motifs of classes 1c and 2^13^; (iii) the classes 1b and 1d can be found only in a few NES sequences, and the majority of the class 1d sequences can be overlapped to the class 1a pattern in the validated NES sets^9,13^; and (iv) reverse(−) of classes 3 and 4 appears to lack β-strands to hydrogen bond with the Lys residue and may not be ideal NES motifs^14^. This empirical class priority is defined as follows: (i) class 1a with five Φs (c1a-5) as priority 1; (ii) class 1 with four Φs (c1a-4), classes 1a-R, 2, 3, and 4 as priority 2; (iii) classes 1a/1c with Thr or Ala in one of their Φ1 or Φ2 positions as priority 3; (iv) classes 1b, 1d, 1c-reverse, and classes 2/3 with Thr or Ala in one of their Φ1 or Φ2 positions as priority 4, and (v) classes 1b/1d with Thr or Ala in one of their Φ1 or Φ2 positions as priority 5. The extracted regions are from the one residue before Φ0 to the two more residues after Φ4 (or shorter if located at the protein C- or N-termini). If the Φ2-Φ4 portion of the extracted region overlaps with experimental evidence (annotated as “mutations that affect nuclear export,” “mutations that affect CRM1 binding,” or “functional export signal” in NESdb, or annotated as “sites” in validNES), it is considered as a candidate NES. If not, it is deemed as a false positive.

### Calculation of disorder propensity and definition of ordered regions

The disorder propensity of the cargo protein sequences is calculated using three different programs, DISOPRED3^25^, SPOT-disorder^26^, and IUPred2A^27^. For DISOPRED3 and SPOT-disorder calculation, which is based on multiple sequence alignment, uniref90_2015_01^34^ database is used to find homologs during PSI-BLAST search^35^. In order to define ordered regions with high confidence, we applied strict cutoff values (~0.1) to decide the order/disorder border lines (note that the default values for disordered regions of these three programs here are ~0.5). If a residue’s disorder propensities predicted by both DISOPRED and SPOT-disorder are below 0.1, the residue is defined as ordered (“O”). If not, the residue is recorded as potentially disordered (“D”). The predicted values by IUPred2A is also recorded for the reference. The sequence segment’s location is determined by scanning the portion of “D” or “O” in the segment and flanking residues (20 residues at both sides) (Fig. S2A). If the portion of “D” mark is more than 90% for the segment and flanking regions, the location of the segment (loc_DISO) is defined as an ordered region (“ORD”). If “O” is more than 90%, the location is determined as a disordered region (“DISO”). The other segments are considered as the ones located in the “boundary” region. The segments in the boundary regions can be found at the end of the ordered regions, or they can locate in the ordered regions where some portions (>10%) have higher disorder propensity than the cutoff value.

### Extraction of the conserved domain information of the cargo proteins

By using the Batch CD-search tool^36^, the conserved domain information for the cargo protein sequences was extracted. Four different databases, i.e., CDD (cdd v3.16), NCBI_Curated (cdd_ncbi v3.16), Pfam (oasis_pfam v3.16), SMART (oasis_smart v3.16), were searched with the expect value threshold of 0.01. The results were retrieved by the Concise mode.

### Prediction of secondary structure

Secondary structures of the cargo protein sequences are predicted by PSIPRED Version 3.21^37^. During PSI-BLAST search^35^ to find homologs, uniref90_2015_01^34^ database is used. In the online table, the confidence level of the prediction is also colored by a gradient from dark (high confidence) to light (low confidence).

### Relative binding energy (E_bind_) prediction

Ten crystal structures of CRM1 bound to various NES peptides, including MVM-NS2 (PDB ID: 6CIT^31^), super PKI (unpublished data), FMRP-1b (5UWO^14^), SNUPN (3GB8^12^), FMRP (5UWJ^14^), SMAD4 (5UWU^14^), HIV-Rev (3NBZ^11^), X11L2 (5UWS^14^), and CPEB4 (5DIF^13^), were utilized as templates. For the CRM1 part, we extracted the residues from 479 to 655 (numbered in scCRM1) to reduce the computation time. For potential NES peptides, the positions from Φ0–1 to Φ4 + 2 positions were modeled (or a shorter segment in case a sequence used in the experimental K_D_ measure is shorter). A given peptide sequence is fitted to the backbone coordinates of every template structure. By using the Rosetta backrub module^38^, the backbone conformations of the fitted NES peptide and the surrounding helices in CRM1 are sampled to generate 50 models (50,000 backrub Monte Carlo trials/steps were run for each model). Among them, five complex structures with the lowest energy are selected and then optimized by the Rosetta relax module^39,40^, which searches the local conformational space around the starting structure. The relaxation was carried out 50 times for each model (i.e., the total number of models for a given peptide sequence is 10 × 50 = 500 models) with ‘-use_input_sc -ex1 -ex2’ flag for more rigorous search. The backrub-modeled backbone conformation was constrained during the relaxation by applying ‘-constrain_relax_to_start_cords’ flag. Structures of the CRM1 protein itself and the free peptide are also modeled separately with the same process. The all-atom energy function REF15 in Rosetta v.3.9 were utilized for all calculation.

The binding energy (E_bind_) is calculated as E_complex_ − E_protein_ − E_peptide_. The values for E_complex_, E_protein_, and E_peptide_ are the average of the lowest 10 energy values among the 500 models. For E_peptide_, we utilized the lowest E_peptide_ among the all different backbone fitted models. Among the various template-fitted models, the one with the lowest E_bind_ score is selected. The E_bind_ scores were corrected with a solvent accessibility term calculated by the NACESS v.2.1.1 program^32^, which calculates the atomic accessible surface defined by rolling a probe of given size around a vdw surface. To penalize the cavity at the interface of CRM1 and low-affinity binders (such as PKI double mutant), the RSA values for the hydrophobic residues at the interface (Fig. S3) were extracted and added to the E_bind_ scores with the optimized weight.

## Supplementary information

Supporting information

## Data Availability

The datasets generated during and/or analyzed during the current study are included in this published article and available via: http://prodata.swmed.edu/nes_pattern_location/.
