# Supplementary material for: Structural prerequisites for CRM1-dependent nuclear export signaling peptides: accessibility, adapting conformation, and the stability at the binding site
Source: Sci Rep. 2019 Apr 29;9:6627. doi: 10.1038/s41598-019-43004-0 (PMC6488578; doi:10.1038/s41598-019-43004-0)
Supplement: Supplementary file 1 — Supporting information [file 41598_2019_43004_MOESM1_ESM.pdf]

Supporting information

Structural prerequisites for CRM1-dependent nuclear export signaling peptides: accessibility, adapting conformation, and the stability at the binding site

Yoonji Lee,[1] Jimin Pei,[2] Jordan M. Baumhardt,[3] Yuh Min Chook,[3] and Nick V. Grishin*,[1,2]

[1]Department of Biophysics, University of Texas Southwestern Medical Center, Dallas, TX 75390, USA. [2]Howard Hughes Medical Institute, University of Texas Southwestern Medical Center, Dallas, TX 75390, USA. [3]Department of Pharmacology, University of Texas Southwestern Medical Center, Dallas, TX 75390, USA.

*Correspondence to: grishin@chop.swmed.edu (NV Grishin)

Table of Contents

Table S1. NES candidate motifs located in the highly ordered region ---------- S3

Table S2. NES candidate motifs which have β-strand components in the middle ---------- S4

Table S3. Number of positive and negative cases of the NES prediction results ---------- S5

Table S4. Performance comparison of E_{bind} score to other sequence-based predictors ---------- S6

Figure S1. Distribution of the classes ---------- S7

Figure S2. Location decision with respect to the disordered or ordered domain and the conserved domain ---------- S7

Figure S3. Correction with the residue solvent accessibility (RSA) ---------- S8

Figure S4. Predicted NES motifs of MEK1 (uniprot ID: Q05116) using Ebind, locNES, netNES, and NESmapper ---------- S8

Figure S5. Predicted NES motifs of p73 (uniprot ID: O15350) ---------- S9

Figure S6. Predicted NES motifs of ADAR1 (uniprot ID: P55265) ---------- S10

Figure S7. Predicted NES motifs of PKI (uniprot ID: P61925) ---------- S10

Figure S8. Predicted NES motifs of MVM (uniprot ID: Q83414) ---------- S11

Figure S9. Predicted NES motifs of STRAD (uniprot ID: Q7RTN6) ---------- S11

Figure S10. Predicted NES motifs of HDAC5 (uniprot ID: Q9UQL6) ---------- S12

Figure S11. Predicted NES motifs of SNUPN (uniprot ID: O95149) ---------- S12

Figure S12. Predicted NES motifs of HPV16E7 (uniprot ID: P03129) ---------- S13

Figure S13. Predicted NES motifs of HIV-REV (uniprot ID: P69718) ---------- S13

Figure S14. Predicted NES motifs of hRio2 (uniprot ID: Q9BVS4) ---------- S14

Figure S15. Predicted NES motifs of CPEB4 (uniprot ID: Q17RY0) ---------- S14

Figure S16. Predicted NES motifs of mDia2 (uniprot ID: Q9Z207) ---------- S15

Figure S17. Predicted NES motifs of CDC7 (uniport ID: O00311) ---------- S15

Figure S18. Predicted NES motifs of FMRP (uniprot ID: Q06787) ---------- S16

Figure S19. Predicted NES motifs of Smad4 (uniprot ID: Q13485) ---------- S16

Figure S20. Predicted NES motifs of X11L2 (uniprot ID: O96018) ---------- S17

Table S1. NES candidate motifs located in the highly ordered region.

name	start#	sequence	secondary structure	†loc_DISO	‡loc_CDD	reference DB
ACT1	167	AGFSLPHAILRIDLAG<br>++++++++++++	CCCCCCCHHHHHCCCC	ORD	MID|NBD_sugar-kinase_HSP70_actin superfamily	validNES:P009
BPV-E1	405	QNIELITFINALKLWL<br>+++++*+++*++	CCCCHHHHHHHHHHHH	ORD	MID|PHA02774	NESdb:139<br>validNES:P187
BPV-E1	405	QNIELITFINALKLWL<br>+++++*+++*++	CCCCHHHHHHHHHHHH	ORD	MID|PHA02774	NESdb:139<br>validNES:P187
E4-34kD	82	DMVLTREELVILRK<br>+++++++*+*	CCCCCHHHHHHHHC	ORD		NESdb:17
FAK	514	RKYSLDLASLILYA<br>++++*+*++*+*++	CCCCCCHHHHHHHH	ORD	MID|PTKc_FAK	NESdb:83
Fbxo7	322	QALNLPDVFGLVVLPL<br>*++*+*	HHCCCCCCCCCCCCCH	ORD	boundary|PI31_Prot_N superfamily	NESdb:230
Fbxo7	325	NLPDVFGLVVLP<br>*++*+	CCCCCCCCCCCC	ORD	boundary|PI31_Prot_N superfamily	NESdb:230
Fbxo7	333	VVLPLELKLRIFRLLD<br>++*+*++*+*	CCCCHHHHHHHHCCCC	ORD	boundary|PI31_Prot_N superfamily	NESdb:230
HDAC1	149	GFCYVNDIVLAILE<br>* *	CCCHHHHHHHHHHH	ORD	MID|HDAC1	NESdb:252
HDAC1	149	GFCYVNDIVLAILELLK<br>* * *	CCCHHHHHHHHHHHHHH	ORD	MID|HDAC1	NESdb:252
HDAC1	152	YVNDIVLAILELLK<br>* * *	HHHHHHHHHHHHHH	ORD	MID|HDAC1	NESdb:252
HDAC1	152	YVNDIVLAILELLK<br>* * *	HHHHHHHHHHHHHH	ORD	MID|HDAC1	NESdb:252
hMSH4	786	AFTLFATHFLELCH<br>*	CEEEEECCCHHHHH	ORD	MID|MutS	NESdb:185
hTERT	966	AGRNMRRKLFGVLRLKC<br>........*...*.*..	HHHHHHHHHHHHHHHCC	ORD	boundary|TERT	NESdb:53<br>validNES:P049
Lgals3	238	RMKNLREISQLGISG<br>++++++++++++	CCCCCCCCCEEEEEC	ORD	boundary|Gal-bind_lectin	validNES:P086
MALT1	365	DVYELTNLLRQLDFKV<br>++++++++	HHHHHHHHHHHCCCEE	ORD	MID|Peptidase_C14	validNES:P198
MoKA; Fbxo38	183	LKIPIGAKIQTLHLVG<br>*+*++	CCCCCCCCEEEEEEEC	ORD		NESdb:202<br>validNES:P197
NOA1	220	YMVNLLDLPDALLPDL<br>+++++++++++	EEEEECCCCCCCCCCC	ORD	MID|RbgA superfamily	NESdb:265
NPM1	83	VQPTVSLGGFEITP<br>*++	CCCCEECCCCEECC	ORD	boundary|Nucleoplasmin	NESdb:93<br>validNES:P082
Nr1i3	240	VHVGFQYEFLELIIHF<br>++++++++++	HHCCCHHHHHHHHHHH	ORD	MID|NR_LBD superfamily	validNES:P194
NURR1	435	QDLLFESAFLELFVLR<br>++*++*+	HHHHHHHHHHHHHHHH	ORD	MID|NR_LBD_Nurr1	NESdb:246
NURR1	435	QDLLFESAFLELFVLRL<br>++*++*+*	HHHHHHHHHHHHHHHHH	ORD	MID|NR_LBD_Nurr1	NESdb:246
NURR1	440	ESAFLELFVLRLAY<br>++*++*+*+	HHHHHHHHHHHHHH	ORD	MID|NR_LBD_Nurr1	NESdb:246
UL47	481	ERYALSAYLTLFVALAE<br>+++++++++++	HHHHHHHHHHHHHHHHH	ORD	MID|Herpes_UL47 superfamily	validNES:P094
Rsp5	683	ELELLIGGIAEIDIED<br>...........*.*..	HHHHHHHCCCCCCHHH	ORD	MID|HUL4	NESdb:229
Smurf1	601	ELELIIGGLDKIDLND<br>+++++++++*+*	HHHHHHHCCCCCCHHH	ORD	MID|HECTc	NESdb:58
Tax	187	PYKRIEELLYKISLTT<br>+++++++++++**++	CHHHHHHHHHHHHHCC	ORD	MID|Tax	NESdb:84<br>validNES:P081
Tax	194	LLYKISLTTGALIILPE<br>+++++**++	HHHHHHHCCCCEEEECC	ORD	boundary|Tax	NESdb:84<br>validNES:P081
Tax	194	LLYKISLTTGALIILP<br>+++++**++	HHHHHHHCCCCEEEEC	ORD	boundary|Tax	NESdb:84<br>validNES:P081

†location of the segment with respect to the ordered (ORD) region
‡location of the segment with respect to the known domains in CDD

Table S2. NES candidate motifs which have β-strand components in the middle.

name	start#	sequence	secondary structure	reference DB	available structures
Cdc6	309	SHLVLIGIANTLDLTD<br>+*+*++	CCEEEEEEEECCCCCCC	NESdb:260	1fnn.1.A; 162-547; Identity: 20.22%<br>β-strand in model
COMMD1	138	IHTPVAIIELELGK<br>+++++++*++*++*++	CCCCEEEEEEEEECC	NESdb:117	no available structure
COMMD1	155	ESEFLCLEFDEVKVNQ<br>++++++	CCCCEEEEEECHHHHHH	NESdb:117	no available structure
COMMD1	157	EFLCLEFDEVKVNQ<br>++++++	CCEEEEEECHHHHHH	NESdb:117	no available structure
Crk	260	VGELVKVTKINVSG<br>** *	CCCEEEEEEEECCCC	NESdb:57	2eyz; 1-304<br>β-strand in NMR
Dengue Virus NS5	334<br>(2825)	VVPMVTQMAMTD<br>**..*.......	CCCCEEEEEEECC	NESdb:78	5ccv.2.A; 2498-3375; Identity: 79.73%<br>small helix in model
FAK	91	RSEEVHWLHVDMGVSS<br>++++*+++*+*+++++	CCCCEEEEECCCCCHHH	NESdb:83	4ny0; 34-405<br>β-strand in x-ray
Hsc70/Hsc54; HSPA8	384	KSENVQDLLLLDVTP<br>*+*++	CCCCCCCEEEEEECCC	NESdb:67<br>validNES:P084	5e84.1.A; 4-609; Identity: 66.11%<br>β-strand in model
β-Arrestin-2	384	DDDIVFEDFARLRLKG<br>+++*++++*+++*+*++	CCCEEEEEECCCCCCCC	NESdb:241	3p2d.1.A; 6-394; Identity: 95.42%<br>β-strand in model
IPMK	170	LMEEIGFLVLGMRVYH<br>++++*+++*+*+++++++	CCCCCCEEEEEEEEEEE	NESdb:253<br>validNES:P212	5w2i; 65-262<br>β-strand in x-ray
LANA2	547	EQFDMVPLVIKLRLRS<br>*+++++++++	CCCCCCCEEEEEEEEEE	NESdb:218<br>validNES:P134	no available structure
LANA2	551	MVPLVIKLRLRS<br>*+++++++++	CCCEEEEEEEEE	NESdb:218<br>validNES:P134	no available structure
MoKA; Fbxo38	190	KIQTLHLVGVNVPE<br>*+*++++++	CEEEEEEEECCCCCC	NESdb:202<br>validNES:P197	no available structure
Nibrin	649	PRKLLLTEFRSLVVSN<br>....**.....*.*..	CCCEEEEEEEEEEEECC	NESdb:111	no available structure
Nibrin	650	RKLLLTEFRSLVVSNH<br>...**.....*.*...	CCEEEEEEEEEEEECCC	NESdb:111	no available structure
NOA1	213	PGPALVLYMVNLLDLPD<br>+++++	CCCCEEEEEEEEECCCCC	NESdb:265	no available structure
NOA1	214	GPALVLYMVNLLDLPD<br>+++++	CCCEEEEEEEEECCCCC	NESdb:265	no available structure
NPM1	38	NEHQLSLRTVSLGA<br>*+*++++++	CCCEEEEEEEEECCC	NESdb:93<br>validNES:P082	5ehd; 14-124; homo-5-mer<br>β-strand in x-ray
NPM1	91	GFEITPPVVLRLKC<br>*+++*+++*+*	CCEECCCEEEEEEE	NESdb:93<br>validNES:P082	5ehd; 14-124; homo-5-mer<br>β-strand in x-ray
nsP2 (VEE)	515<br>(1050)	VLNQLCVRFFGLDLDS<br>+++++*+*++	HHCCEEEEEECCCCCCC	NESdb:92<br>validNES:P190	5ezq.1.A; 1003-1322; Identity: 98.51%<br>helix in model
p21Cip1; CDKN1A	60	EGDFAWERVRGLGLPK<br>+++*+*++	CCCCEEEEEECCCCCCC	NESdb:210<br>validNES:P143	1jsu.1.C; 14-80; Identity: 39.71%<br>β-strand in model
Protein 9b	41	KVYPIILRLGSQLSLSM<br>+*+*+++*+*	CEEEEEEEEECCCCHHHH	NESdb:214<br>validNES:P099	2cme; 9-25<br>β-strand in x-ray
Protein UL84	222	RMAIVRLSLNLFALRI<br>*+*++++++++	HHCEEEEEEEEEEEEEEC	NESdb:209<br>validNES:P087	no available structure
Protein UL84	224	AIVRLSLNLFALRIIT<br>*+*++++++++	CEEEEEEEEEEEEECCC	NESdb:209<br>validNES:P087	no available structure
Protein UL84	355	SPPDLTLSSLTLYQ<br>*+++++++	CCCCCEEEEEEEEEE	NESdb:209<br>validNES:P087	no available structure
Sirt1	423	DEVDLLIVIGSSLKVRP<br>* *****	CCCCEEEEEECCCCCCCCC	NESdb:264	5btr.1.A; 166-505; Identity: 97.48%<br>β-strand in model
Smad1	402	TVYELTKMCTIRMSF<br>*+++*+++++	EEEECCCEEEEEEEEE	NESdb:40<br>validNES:P032	5zok; 266-462; heteromer<br>β-strand in x-ray
Smad1	403	VYELTKMCTIRMSF<br>*+++*+++++	EEECCCEEEEEEEE	NESdb:40<br>validNES:P032	5zok; 266-462; heteromer<br>β-strand in x-ray
Smad1	405	ELTKMCTIRMSFVK<br>*+++*+++++	ECCCEEEEEEEEEE	NESdb:40<br>validNES:P032	5zok; 266-462; heteromer<br>β-strand in x-ray
SMARCB1	262	VIIKLNIHVGNISLVD<br>+++++++++++	EEEEEEEEECCEEEEEE	validNES:P218	5l7b.1.A; 259-319; Identity: 39.34%<br>β-strand in model
Stau2	1	XMLQINQMFSVQLSLGE<br>*+++++++*++	CCCCCCEEEEEEEEECCE	NESdb:122	no available structure
Stau2	1	XMLQINQMFSVQLSLGE<br>+++++++++++	CCCCCCEEEEEEEEECCE	validNES:P139	no available structure
TDP-43	244	CGEDLIIKGISVHI<br>***	HCCCEEECCEEEEE	NESdb:193	4bs2; 102-269<br>β-strand in NMR

TDP-43	246	`EDLIIKGISVHISNAE`<br>`  ***`	`CCEEECCEEEEEEECC`	NESdb:193	4bs2; 102-269<br>β-strand in NMR
VP3	29	`HCREIRIGIAGITITL`<br>`        ++++++++`	`EEEEEEEEEEEEEEEE`	NESdb:134<br>validNES:P118	no available structure
VP3	31	`REIRIGIAGITITL`<br>`      ++++++++`	`EEEEEEEEEEEEEE`	NESdb:134<br>validNES:P118	no available structure

Table S3. Number of positive and negative cases of the NES prediction results

protein	# total	# NES	# predicted	# FP	# FN	# TN	# TP	# predicted	# FP	# FN	# TN	# TP	# predicted	# FP	# FN	# TN	# TP	# predicted	# FP	# FN	# TN	# TP
O00311_Cdc7	35	2	2	2	2	31	0	5	4	1	29	1	1	0	1	33	1	3	3	2	30	0
O15350_p73	24	1	2	1	0	22	1	4	3	0	20	1	1	0	0	23	1	3	2	0	21	1
O95149_SNUPN	13	1	2	1	0	11	1	2	1	0	11	1	0	0	1	12	0	0	0	1	12	0
O96018_X11L2	17	1	2	1	0	15	1	2	1	0	15	1	0	0	1	16	0	1	1	1	15	0
P03129_HPV16E7	7	1	1	0	0	6	1	3	2	0	4	1	1	0	0	6	1	0	0	1	6	0
P55265_ADAR1	22	1	3	2	0	19	1	4	3	0	18	1	0	0	1	21	0	3	2	0	19	1
P61925_PKI	3	1	1	0	0	2	1	1	0	0	2	1	1	0	0	2	1	1	0	0	2	1
P69718_HIV-REV	6	1	1	0	0	5	1	2	1	0	4	1	1	1	1	4	0	2	2	1	3	0
Q05116_STRAD	29	2	3	1	0	26	2	5	3	0	24	2	1	0	1	27	1	2	0	0	27	2
Q06787_hRio2	14	1	1	0	0	13	1	2	2	1	11	0	0	0	1	13	0	0	0	1	13	0
Q13485_HDAC5	42	1	4	3	0	38	1	4	3	0	38	1	1	1	1	40	0	4	3	0	38	1
Q17RY0_mDia2	73	1	6	5	0	67	1	11	10	0	62	1	0	0	1	72	0	12	12	1	60	0
Q7RTN6_CPEB4	12	1	2	1	0	10	1	2	1	0	10	1	1	1	1	10	0	0	0	1	11	0
Q83414_MEK1	18	1	2	1	0	16	1	2	1	0	16	1	1	0	0	17	1	4	3	0	14	1
Q9BVS4_FMRP	24	1	3	2	0	21	1	3	2	0	21	1	1	0	0	23	1	7	6	0	17	1
Q9UQL6_Smad4	15	1	4	3	0	11	1	3	2	0	12	1	1	0	0	14	1	1	0	0	14	1
Q9Z207_MVM	6	1	1	0	0	5	1	2	1	0	4	1	2	1	0	4	1	1	0	0	5	1
	360	19	40	23	2	318	17	57	40	2	301	17	13	4	10	337	9	44	34	9	307	10

Table S4. Performance comparison of E_{bind} score to other sequence-based predictors.

	E_{bind}	*LocNES*	*NetNES*	*NESmapper*
**TPR (recall, sensitivity)**	**0.895**	**0.895**	**0.474**	**0.526**
FNR (miss rate)	0.105	0.105	0.526	0.474
**TNR (specificity)**	**0.933**	**0.883**	**0.988**	**0.9**
FPR (fall-out)	0.067	0.117	0.012	0.1
LR+	13.27	7.628	40.38	5.279
LR-	0.113	0.119	0.533	0.526
**DOR**	**117.5**	**63.96**	**75.83**	**10.03**
ACC (accuracy)	0.931	0.883	0.961	0.881
PPV (precision)	0.425	0.298	0.692	0.227
**F_1 score**	**0.576**	**0.447**	**0.563**	**0.317**

TPR (true positive rate; recall; sensitivity) = TP/(TP+FN)
FNR (false negative rate; miss rate) = FN/(TP+FN)
TNR (true negative rate; specificity) = TN/(TN+FP)
FPR (false positive rate; fall-out) = FP/(TN+FP)
LR+ (positive likelihood ratio) = TPR/FPR
LR- (negative likelihood ratio) = FNR/TNR
DOR (diagnostic odds ratio) = LR+/LR-
ACC (accuracy) = (TP+TN)/(TP+TN+FP+FN)
PPV (positive predictive value; precision) = TP/(TP+FP)
F_1 score = 2*precision*recall/(precision+recall)

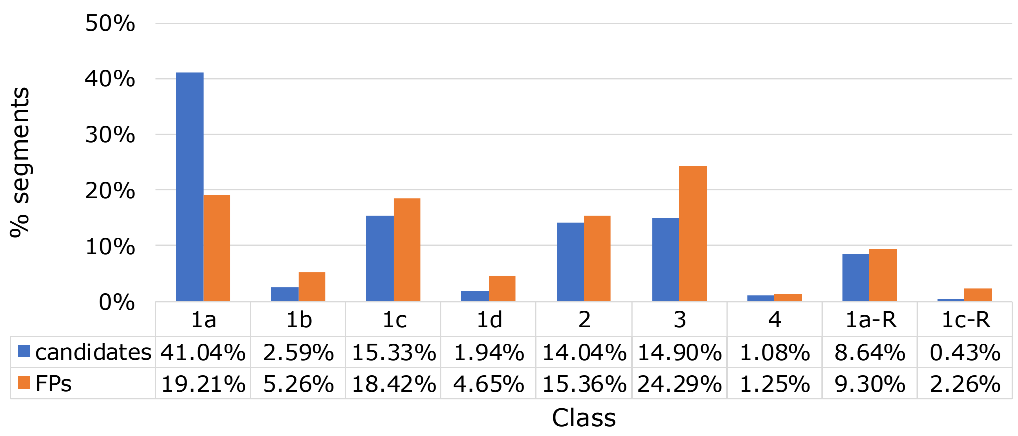

Figure S1. Distribution of the classes.

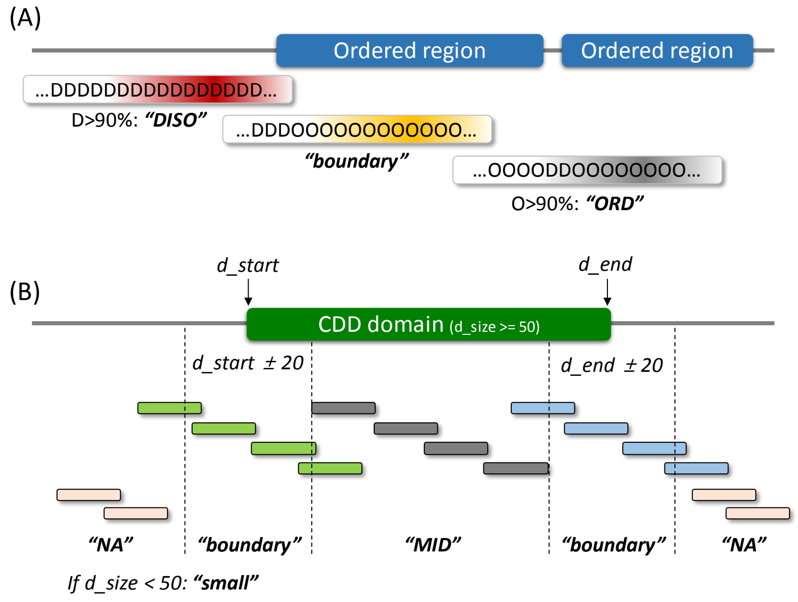

Figure S2. Location decision with respect to (A) the disordered or ordered domain and (B) the conserved domain.

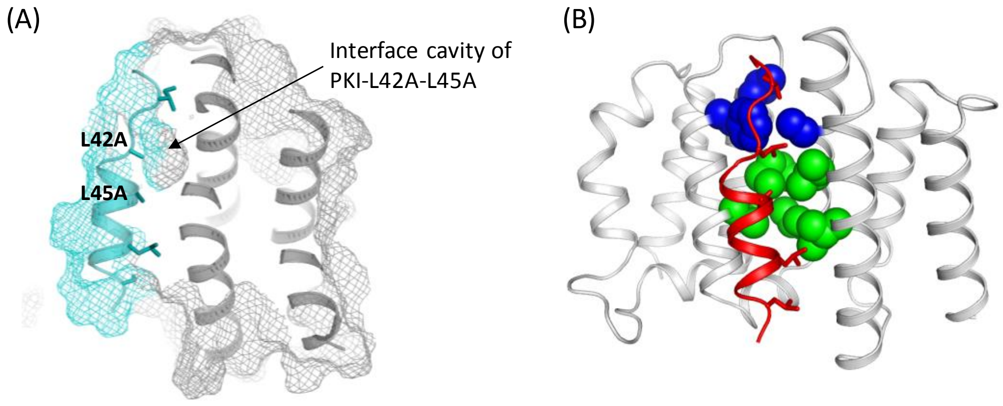

Figure S3. Correction with the residue solvent accessibility (RSA). (A) The internal cavity in the model of CRM1-PKI double mutant. (B) Residues of CRM1 utilized in the RSA calculation. The RSA values for V591, F583, A552 (blue), I555, M556, I532, V576, V559 (green) are extracted and summed. For the short peptides (class 3 such as CDC7 or mDia2), only the residues in green were utilized for RSA calculation.

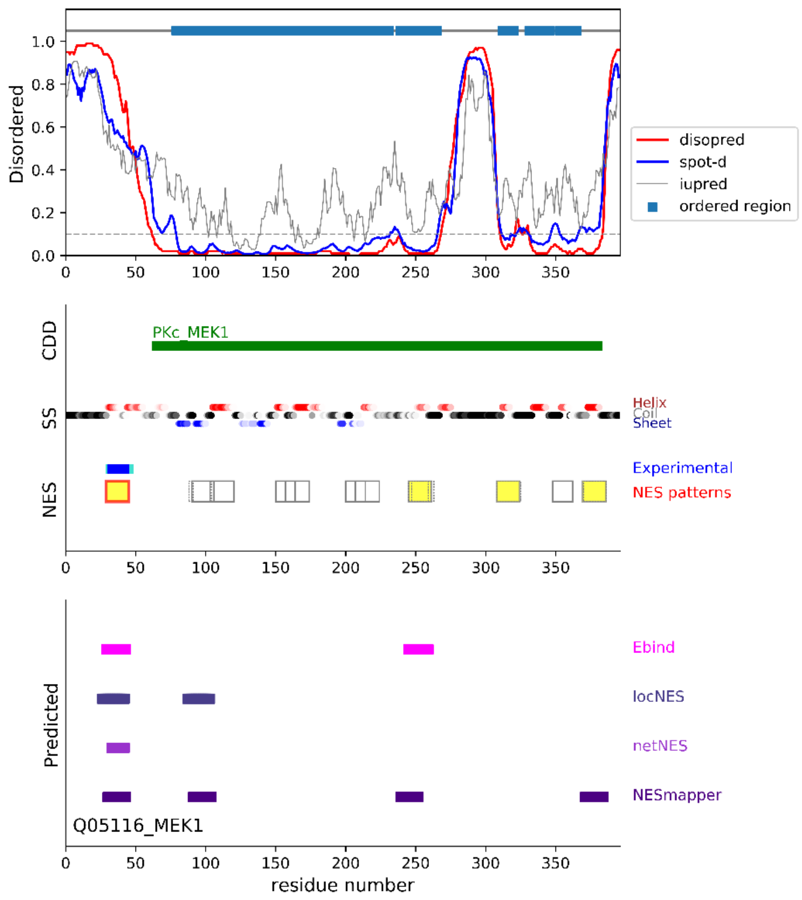

Figure S4. Predicted NES motifs of MEK1 (uniprot ID: Q05116) using E_{bind}, locNES, netNES, and NESmapper. Disordered propensity, known domain information, predicted secondary structure, and the location of the consensus patterns are plotted together. The defined ordered region (by the cutoff value of 0.1; gray dashed line) is represented by the sky-blue box at the top. The regions of the conserved domains annotated in the conserved domain database (CDD) are marked in the middle. The predicted secondary structures (SS) were colored by red, black, and blue for α-

helix, coil, and β-strand, respectively. The gradient of the color corresponds to the confidence level of the prediction. For the NES regions, experimentally validated regions are displayed in blue (with mutation data annotated in NESdb) and cyan (annotated as a functional sequence in NESdb or as a site in validNES). All the consensus pattern matching segments (NES) are marked with black boxes. Segments not in the ordered regions and without β-strand predictions in the middle are highlighted in yellow. The red boxes are the pattern-matching segments overlapping with experimental evidence. The predicted NES motifs using Ebind, locNES, netNES, and NESmapper programs were represented by magenta, slate, violet, and dark purple, respectively, at the bottom.

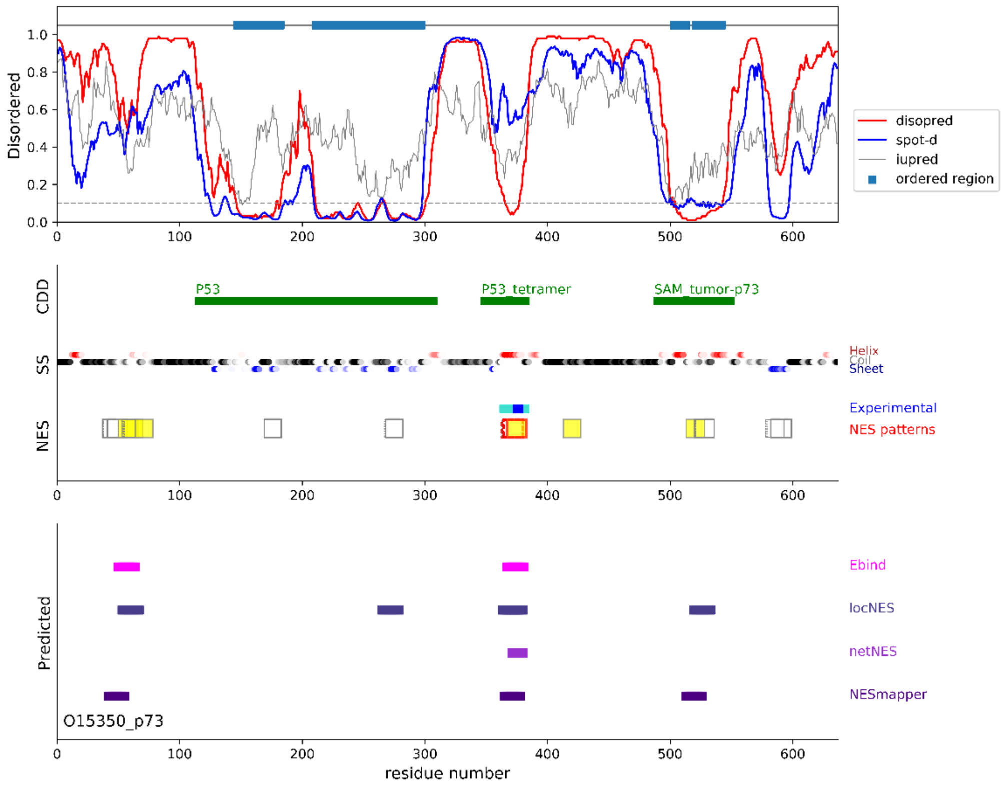

Figure S5. Predicted NES motifs of p73 (uniprot ID: O15350) using E_{bind}, locNES, netNES, and NESmapper.

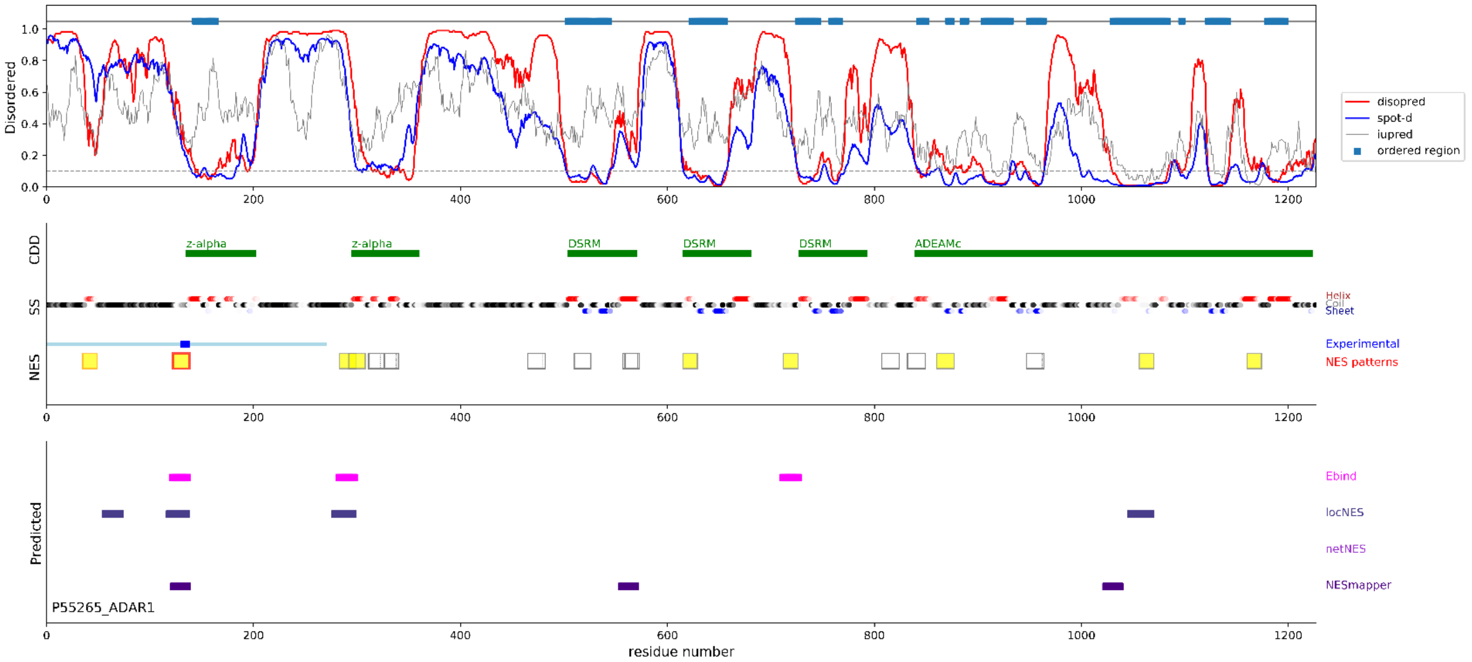

Figure S6. Predicted NES motifs of ADAR1 (uniprot ID: P55265) using E_{bind}, locNES, netNES, and NESmapper.

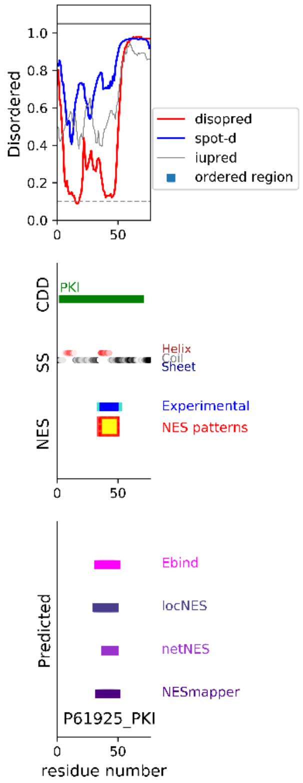

Figure S7. Predicted NES motifs of PKI (uniprot ID: P61925) using E_{bind}, locNES, netNES, and NESmapper.

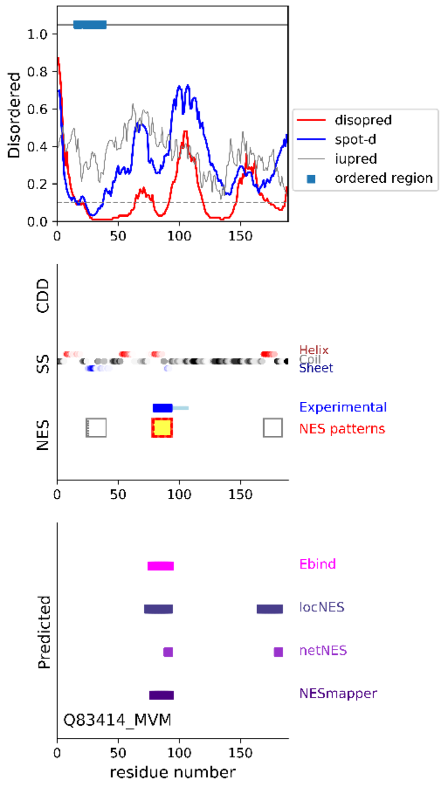

Figure S8. Predicted NES motifs of MVM (uniprot ID: Q83414) using E_{bind}, locNES, netNES, and NESmapper.

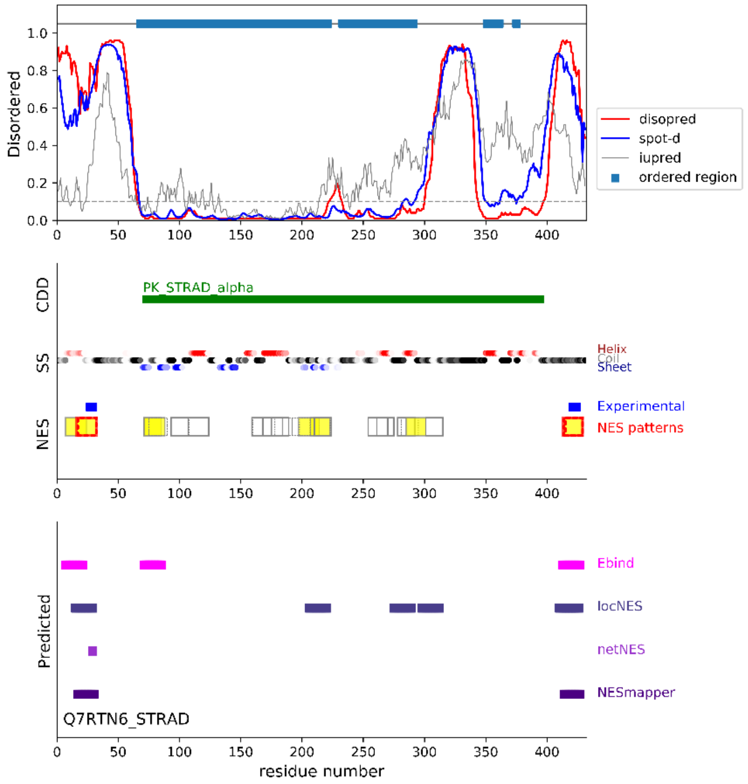

Figure S9. Predicted NES motifs of STRAD (uniprot ID: Q7RTN6) using E_{bind}, locNES, netNES, and NESmapper.

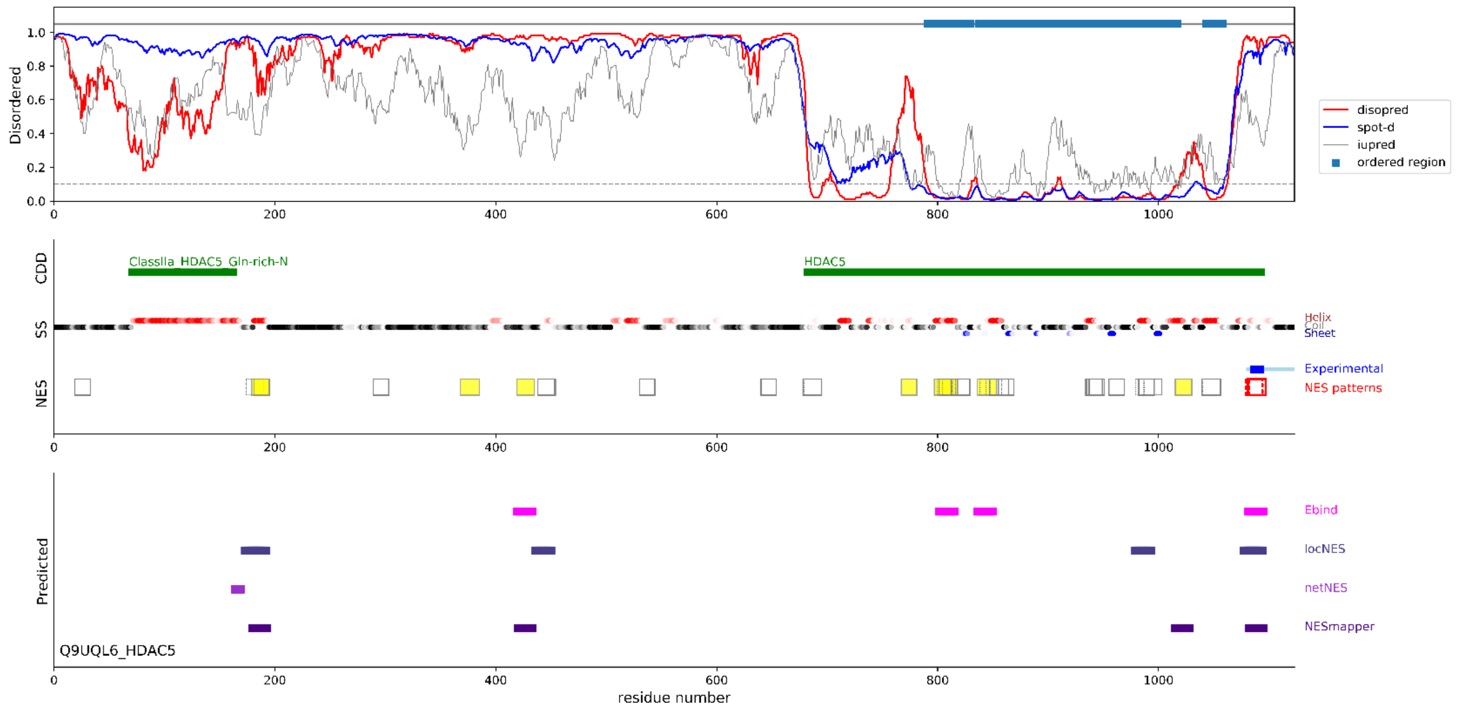

Figure S10. Predicted NES motifs of HDAC5 (uniprot ID: Q9UQL6) using E_{bind}, locNES, netNES, and NESmapper.

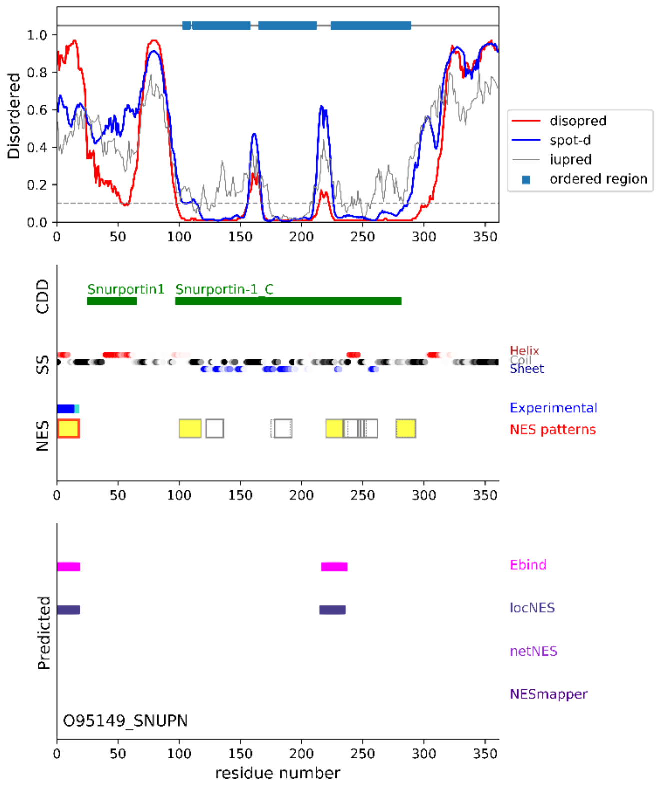

Figure S11. Predicted NES motifs of SNUPN (uniprot ID: O95149) using E_{bind}, locNES, netNES, and NESmapper.

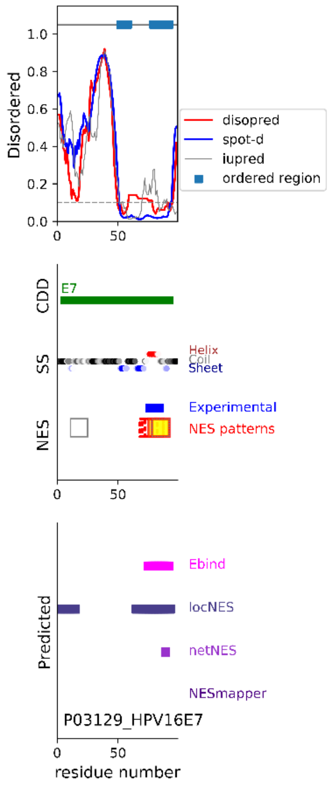

Figure S12. Predicted NES motifs of HPV16E7 (uniprot ID: P03129) using E_{bind}, locNES, netNES, and NESmapper.

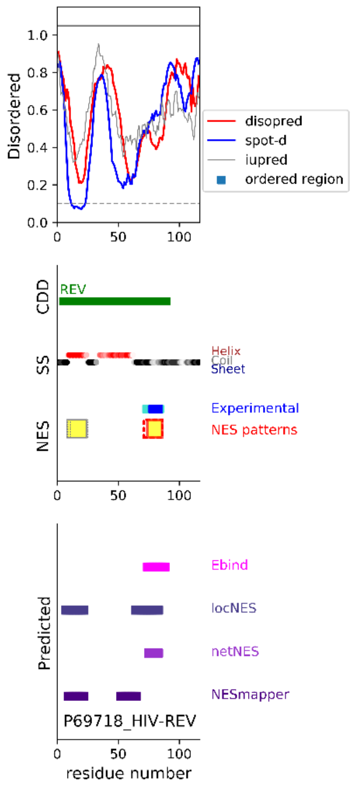

Figure S13. Predicted NES motifs of HIV-REV (uniprot ID: P69718) using E_{bind}, locNES, netNES, and NESmapper.

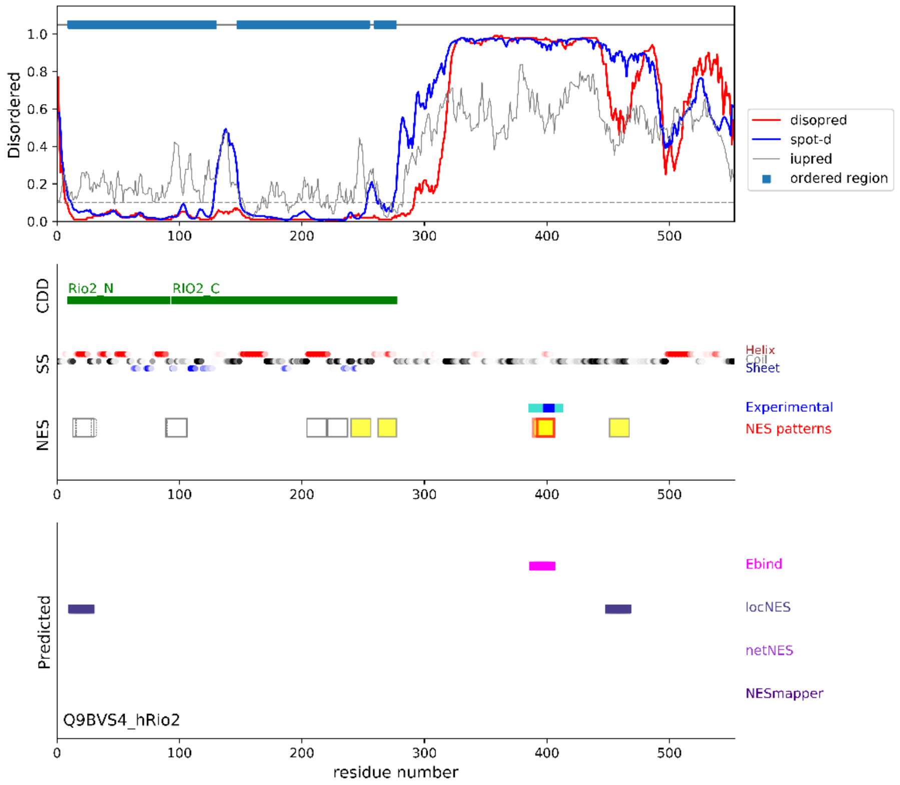

Figure S14. Predicted NES motifs of hRio2 (uniprot ID: Q9BVS4) using E_{bind}, locNES, netNES, and NESmapper.

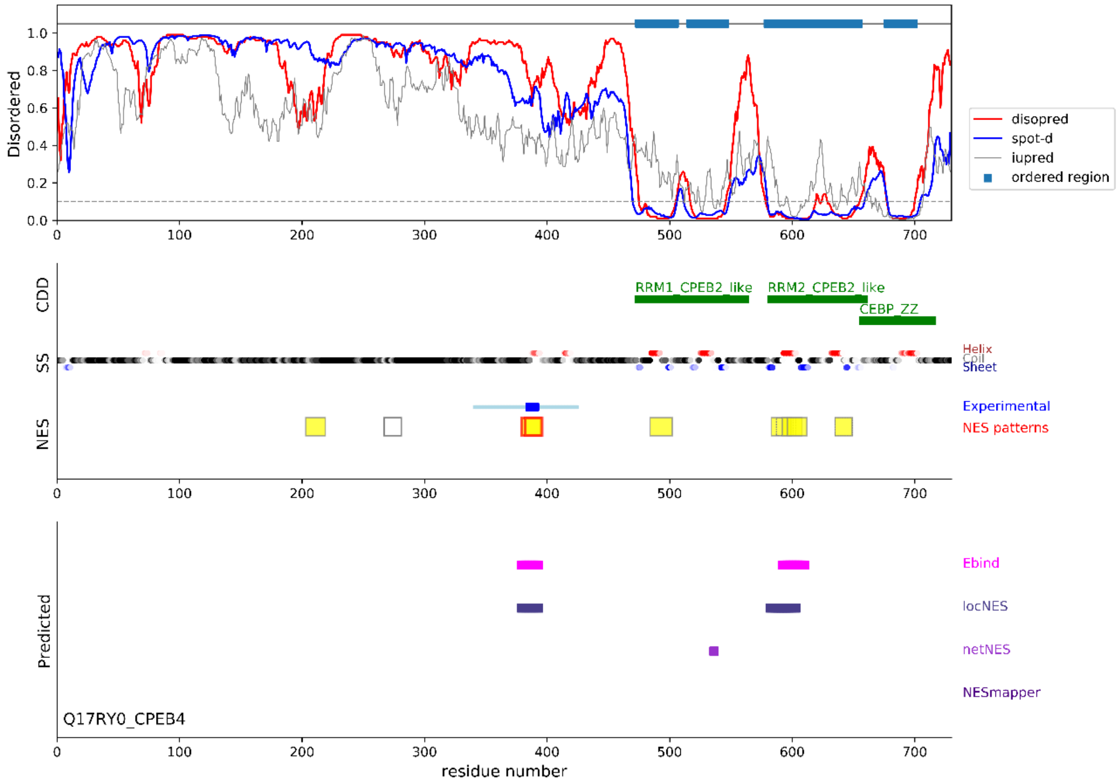

Figure S15. Predicted NES motifs of CPEB4 (uniprot ID: Q17RY0) using E_{bind}, locNES, netNES, and NESmapper.

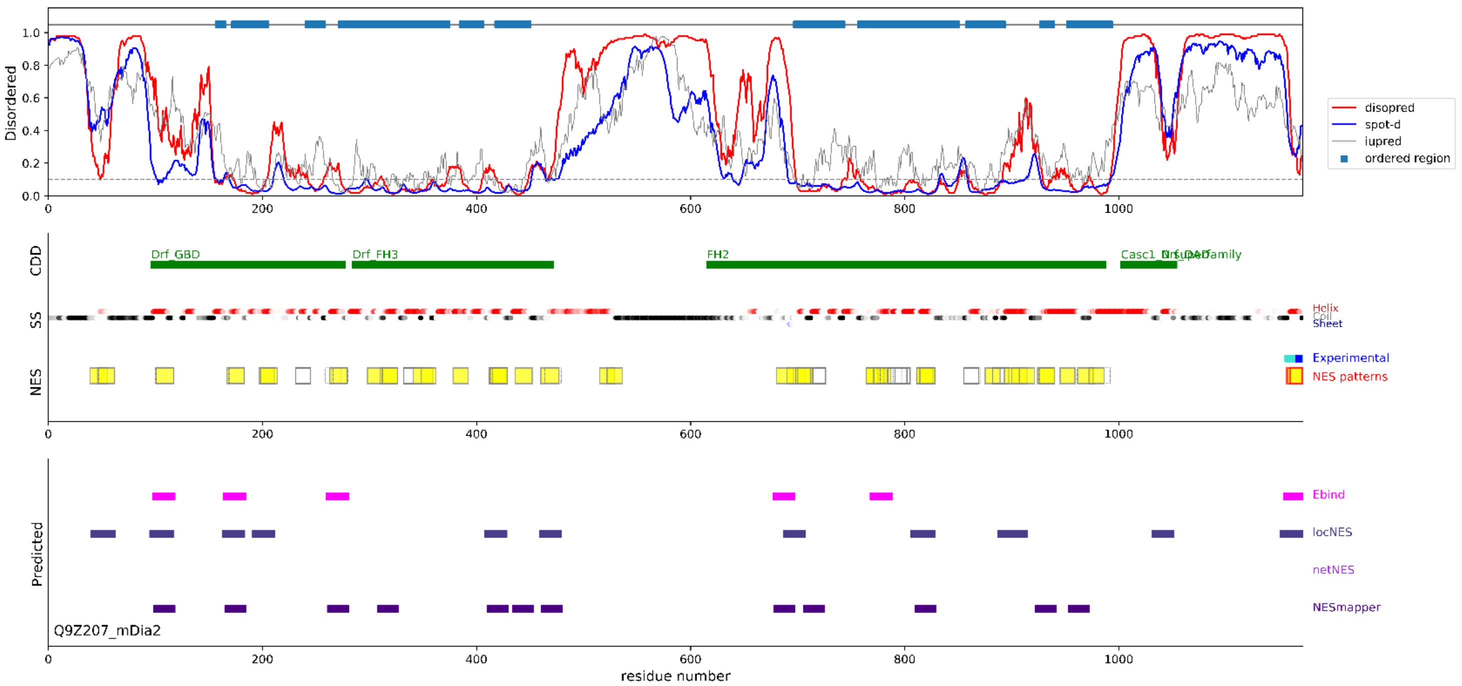

Figure S16. Predicted NES motifs of mDia2 (uniprot ID: Q9Z207) using E_{bind}, locNES, netNES, and NESmapper.

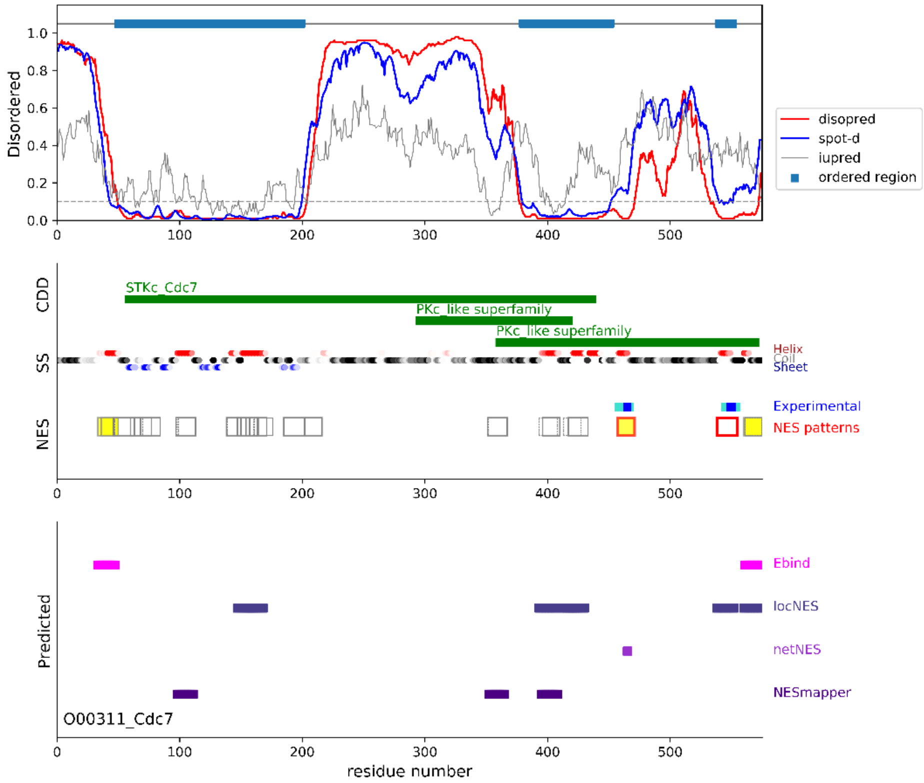

Figure S17. Predicted NES motifs of CDC7 (uniport ID: O00311) using E_{bind}, locNES, netNES, and NESmapper.

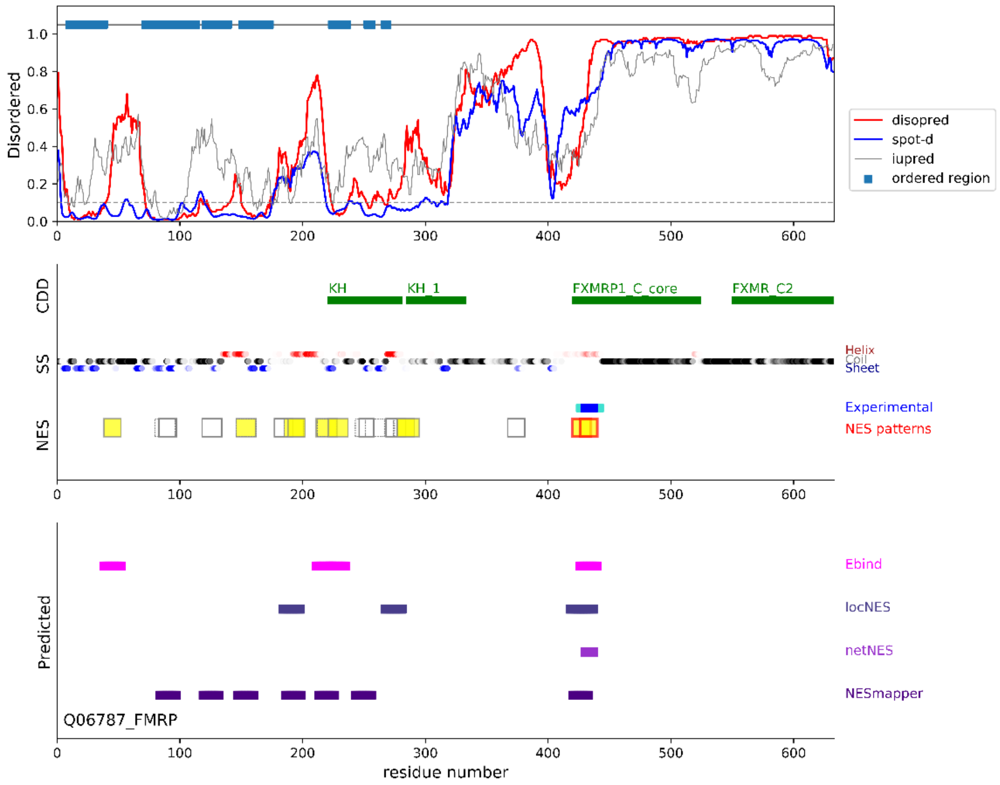

Figure S18. Predicted NES motifs of FMRP (uniprot ID: Q06787) using E_{bind}, locNES, netNES, and NESmapper.

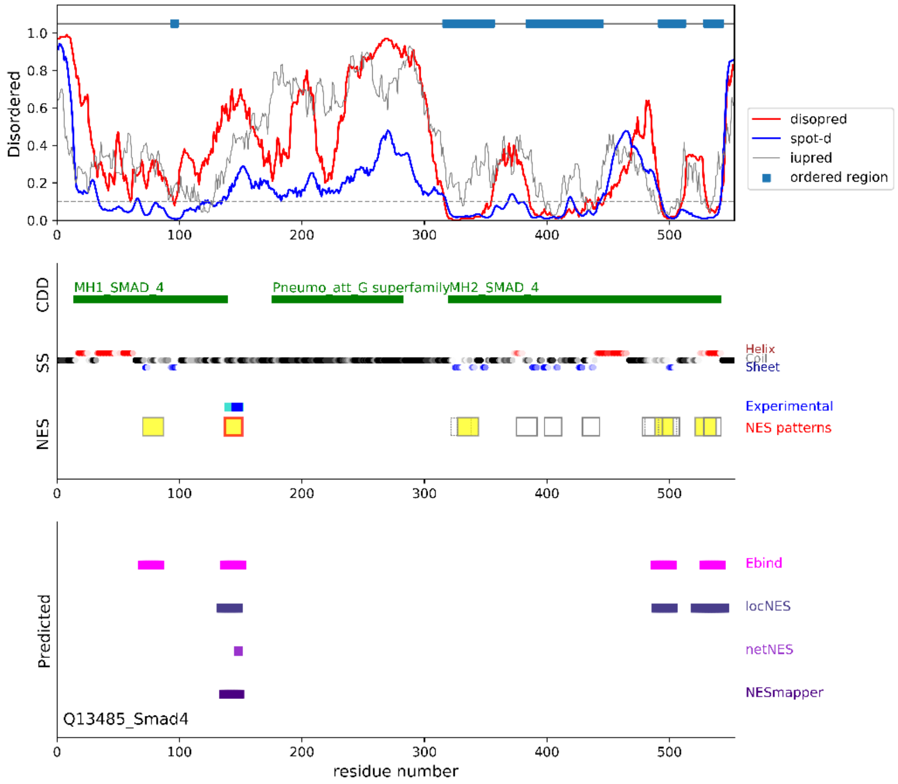

Figure S19. Predicted NES motifs of Smad4 (uniprot ID: Q13485) using E_{bind}, locNES, netNES, and NESmapper.

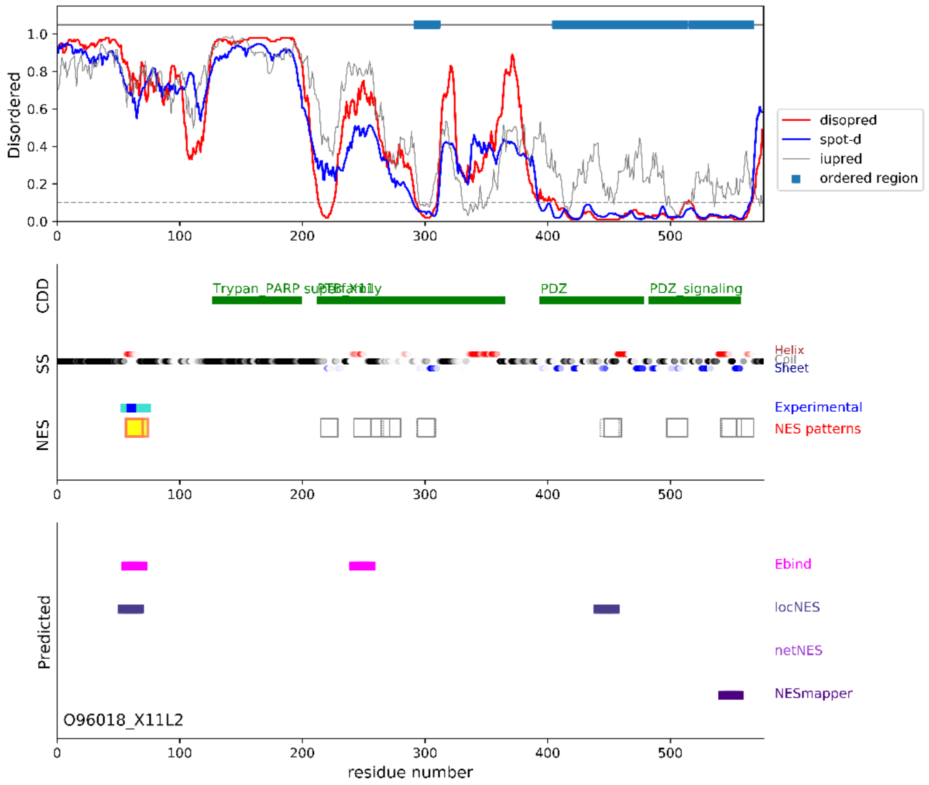

Figure S20. Predicted NES motifs of X11L2 (uniprot ID: O96018) using E_{bind}, locNES, netNES, and NESmapper.
